# Non‐Invasive Diagnosis of Chronic Myocardial Infarction via Composite In‐Silico‐Human Data Learning

**DOI:** 10.1002/advs.202406933

**Published:** 2025-06-19

**Authors:** Rana Raza Mehdi, Nikhil Kadivar, Tanmay Mukherjee, Emilio A. Mendiola, Akila Bersali, Dipan J. Shah, George Karniadakis, Reza Avazmohammadi

**Affiliations:** ^1^ Department of Biomedical Engineering Texas A&M University College Station TX 77843 USA; ^2^ School of Engineering Brown University Providence RI 02912 USA; ^3^ Houston Methodist DeBakey Heart & Vascular Center Houston TX 77030 USA; ^4^ Division of Applied Mathematics Brown University Providence RI 02912 USA; ^5^ School of Engineering Medicine Texas A&M University Houston TX 77030 USA; ^6^ J. Mike Walker '66 Department of Mechanical Engineering Texas A&M University College Station TX 77843 USA; ^7^ Department of Cardiovascular Sciences Houston Methodist Research Institute Houston TX 77030 USA

**Keywords:** cardiac strains, LGE‐CMR, multi‐fidelity, myocardial infarction, UNet architectures

## Abstract

Myocardial infarction (MI) continues to be a leading cause of death worldwide. The precise quantification of infarcted tissue is crucial to diagnosis, therapeutic management, and post‐MI care. Late gadolinium enhancement‐cardiac magnetic resonance (LGE‐CMR) is regarded as the gold standard for precise infarct tissue localization in MI patients. A fundamental limitation of LGE‐CMR is the invasive intravenous introduction of gadolinium‐based contrast agents that present potential high‐risk toxicity, particularly for individuals with underlying chronic kidney diseases. Herein, a completely non‐invasive methodology is developed to identify the location and extent of an infarct region in the left ventricle via a machine learning (ML) model using only cardiac strains as inputs. In this approach, the remarkable performance of a multi‐fidelity ML model is demonstrated, which combines rodent‐based in‐silico‐generated training data (low‐fidelity) with very limited patient‐specific human data (high‐fidelity) in predicting LGE ground truth. The results offer a new paradigm for developing feasible prognostic tools by augmenting synthetic simulation‐based data with very small amounts of in vivo human data. More broadly, the proposed approach can significantly assist with addressing biomedical challenges in healthcare where human data are limited.

## Graphical abstract

This study presents a non‐invasive machine learning model to identify infarct regions in the left ventricle using cardiac strain data. By combining rodent‐based simulated data with limited human data, the model achieves high accuracy in predicting infarct size and location without the need for gadolinium contrast agents, offering a promising alternative to traditional methods.

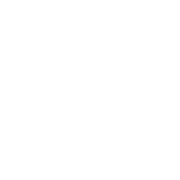

## 1 Introduction

Myocardial infarction (MI), commonly known as a heart attack, is a critical and life‐threatening cardiovascular condition that claimed the lives of approximately one million people in 2016 in the US and continues to be a leading cause of death in the United States.^[^ ^1^, ^2^ ^]^ This acute condition occurs when the blood supply to the cardiac muscle is blocked, leading to the death of the myocardium.^[^ ^3^, ^4^ ^]^ The long‐term consequences of MI can be profound, often leading to chronic cardiac dysfunction and an increased risk of additional subsequent heart attacks.^[^ ^5^, ^6^ ^]^ Despite improvements in post‐infarction survival rates following therapeutic advances,^[^ ^7^ ^]^ MI often leads to chronic heart failure, which is the leading cause of mortality associated with MI.^[^ ^8^, ^9^ ^]^ Scar formation in the myocardium following MI significantly impacts cardiac function,^[^ ^10^ ^]^ with the size and extent of the scar (infarct region) evidently playing an important role in the long‐term outcome of MI.^[^ ^5^ ^]^ Indeed, the precise quantification of infarcted tissue within the whole myocardium has emerged as a crucial part of diagnosis, therapeutic management, and improved post‐MI care.^[^ ^11^ ^]^ Additionally, assessing the extent and location of myocardial infarct remains essential for developing personalized risk stratification and treatment protocols.^[^ ^12^ ^]^

The utilization of cardiac magnetic resonance (CMR) with late gadolinium enhancement (LGE) is regarded as the gold standard for precise infarct tissue localization in MI patients. While this imaging method provides the most precise delineation of MI regions, it is not without limitations and presents several inherent drawbacks within the clinical setting.^[^ ^13^, ^14^, ^15^ ^]^ One notable limitation lies in the risk it imparts upon patients, primarily due to the invasive intravenous introduction of gadolinium‐based contrast agents (GCA) throughout the imaging process.^[^ ^13^ ^]^ This presents a potential high‐risk scenario, particularly for individuals with underlying chronic kidney diseases, as the administration of GCA can have fatal consequences in such patients.^[^ ^13^ ^]^ Moreover, the reliance on CMR imaging for infarct localization undermines the affordability and ubiquity of this procedure, with echocardiography being the safest, most cost‐effective, and ubiquitous cardiac imaging modality. Additionally, LGE‐CMR introduces non‐repeatability and time‐consuming factors into the clinical workflow. The process relies on experienced clinicians to detect and manually segment “bright” areas from the LGE‐CMR images, a step that is susceptible to considerable variation in interpretation among different observers.^[^ ^15^ ^]^ This multi‐step process, which includes LGE in CMR imaging, manual segmentation, and quantification of segmented areas, not only extends the time needed for diagnosis but may also result in cumulative errors.^[^ ^14^ ^]^ In light of these limitations, there is a pressing need to develop alternative, less invasive, and more efficient approaches to infarct localization in MI patients, which can address the shortcomings associated with LGE approach and its reliance on CMR, GCA, and manual segmentation. To overcome the limitations associated with LGE‐CMR imaging, we propose the use of cardiac strains derived from myocardial deformation analysis as a promising alternative to detect and characterize infarct regions without the use of LGE in imaging. Several studies have investigated the ability of cardiac strains to identify MI. For example, Sengupta et al.^[^ ^16^ ^]^ employed speckle‐tracking echocardiography to assess circumferential and longitudinal strains, demonstrating their ability to identify MI‐induced myocardial dysfunction. Similarly, Wu et al.^[^ ^17^ ^]^ utilized CMR tagging to quantify circumferential and radial strains, providing valuable insights into regional myocardial mechanics in MI patients. Moreover, techniques such as feature tracking, strain‐encoded CMR, and displacement encoding with stimulated echoes (DENSE) have been employed to measure various components of cardiac strains, further enhancing the precision of MI characterization.^[^ ^18^, ^19^, ^20^ ^]^ While these studies have provided significant insights into the capacity of cardiac strains as markers to detect infarct myocardium, the need to develop a standardized tool to locate infarct and subcontractile regions in the heart that can be seamlessly integrated into routine clinical use persists.

In this work, we introduce an ML approach to assess the location and extent of MI regions in the left ventricle (LV). The ML model predicts the infarct size and location utilizing circumferential, radial, and longitudinal (CRL) strains, which can be readily extracted from routine CMR images and potentially other cardiac imaging modalities, including echocardiography. We proposed a single‐fidelity ML model based on the UNet architecture^[^ ^21^ ^]^ trained on data from low‐fidelity rodent computational cardiac models (RCCMs). This model was trained using CRL strains at end‐systole (ES) obtained from RCCM simluations, enabling it to predict the location and extent of MI regions. We then extended the single‐fidelity ML model into a composite neural network architecture^[^ ^22^ ^]^ that incorporated multi‐fidelity data (RCCM plus LGE‐CMR human data). This multi‐fidelity modeling approach was used to enhance the predictive accuracy of the ML model by augmenting it with human LGE‐CMR data. Multi‐fidelity methods have gained prominence in recent years for their ability to leverage heterogeneous data sources with different fidelity to improve the overall accuracy and efficiency.^[^ ^23^, ^24^, ^25^ ^]^ These methods are particularly effective in scenarios where high‐fidelity data are very limited and expensive to obtain, while low‐fidelity data are abundant and easily affordable. Often, low‐fidelity data can provide valuable insights into the trends of high‐fidelity data, thus enhancing the predictive accuracy of the multi‐fidelity modeling approach when compared to single‐fidelity modeling, even with a limited set of high‐fidelity data.^[^ ^26^, ^27^, ^28^ ^]^ The multi‐fidelity framework offers significant advantages in terms of effectiveness, as it can model complex correlations between data of varying fidelity.^[^ ^29^ ^]^ The trained ML model can seamlessly integrate with the CMR image processing pipeline, extracting strains from raw CMR images, which then serve as inputs to the ML model for infarct region prediction. Our findings indicate that the proposed ML model is proficient at learning the complex relationship between cardiac strains and infarct labels, making it a promising tool for infarct region estimation.

## 2 Experimental Section

### 2.1 Overview

Subject‐specific finite‐element (FE) RCCMs was developed by integrating in vivo hemodynamic data with ex vivo mechanical and imaging data obtained from infarcted hearts. The development of the RCCMs is briefly described in the following subsection, and a comprehensive, detailed description of the RCCM development is reported in Mendiola et al.^[^ ^30^ ^]^ Moreover, a library comprising of 592 RCCMs was developed by varying infarct sizes and stiffness levels. Extensive simulations were conducted to generate a large diverse dataset used to train and test the proposed ML models. Finally, the trained models were evaluated against human LGE‐CMR data to further assess the efficacy and accuracy of the single‐fidelity and multi‐fidelity approaches.

### 2.2 Rodent Cardiovascular Computational Model Simulations

#### 2.2.1 Animal Model of Myocardial Infarction

The procedures performed on the animals in this study followed protocols that adhered to the ethical treatment guidelines approved by the Institutional Animal Care and Use Committee (IACUC) at the Texas Heart Institute (THI). Four male Wistar‐Kyoto (WKY) rats, aged 8 weeks at the beginning of the experiment, were utilized. Anteriobasal infarct was induced through ligation of the left anterior descending artery near the base of the heart. The infarcted rats were sacrificed at four timepoints post‐MI (1‐, 2‐, 3‐, and 4 weeks, n = 1 at each timepoint). Immediately prior to sacrifice, pressure‐volume (P‐V) measurements of the LV were collected; subjects in the infarct group were then given GCA in the tail vein. The heart was excised and flushed with phosphate‐buffered saline. The ventricles were then filled with an octreotide solution to approximately end‐diastolic (ED) pressure. Heart tissues were fixed in a 10% formalin solution. High‐resolution CMR imaging was conducted, including T1‐weighted and diffusion tensor imaging (DTI), and employed to develop FE RCCMs, starting with reconstructing the 3‐D biventricular model as described below. In a parallel animal study,^[^ ^30^, ^31^ ^]^ 24 similar WKY rats (with equal numbers of male and female, n = 6 at each timepoint post‐MI) were used for biaxial mechanical testing. These rats provided passive stiffness values used in Section 2.2.3. The statistical spreads in stiffness values of the healthy and infarct regions for each timepoint were used to provide variations of stiffness (described in Section 2.2.4) across the initial four heart geometries examined in this study.

#### 2.2.2 Infarct Identification and Fiber Architecture Mapping

**Figure 1:**
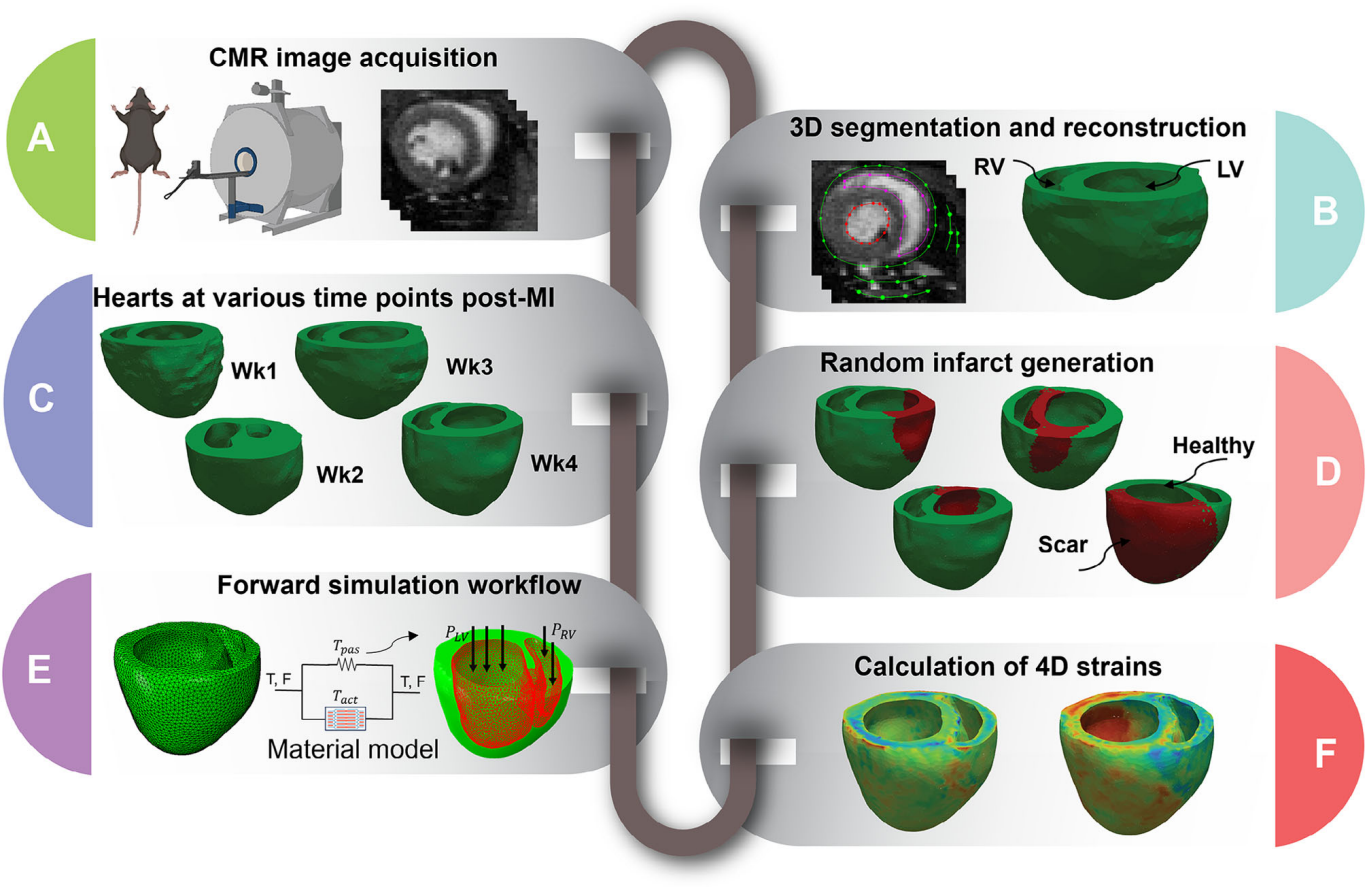
Development of simulated cardiac strains by the use of computational rodent heart models involved reconstruction of 3‐D cardiac geometry from cardiac magnetic resonance (CMR) images (A, B), creation of a library of finite‐element models with random infarct size and location from cardiac geometries at different time points of post‐myocardial infarction (C, D), and estimation of cardiac strains via finite‐element simulation using experimentally derived passive and active properties (E, F).

The 3‐D biventricular heart geometries were reconstructed from CMR scans truncated at the valve plane, and each geometry was discretized using tetrahedral elements (**Figure** **1**). The remote and infarct regions of the post‐MI heart models were determined by identifying areas of the LGE‐CMR that showed increased contrast relative to remote myocardium. The infarct region was also evident in DTI scans as a region with no signal or disturbed fibers. The myofiber orientation distribution in the remote region, reconstructed from DTI scans and weighted by principal directions at each voxel, was registered to the corresponding ventricular mesh from the same rodent heart.

#### 2.2.3 Constitutive Model for the Myocardium

A transversely isotropic hyperelastic material model (characterized by the myofiber direction) was used to capture both passive and active behaviors of the healthy myocardium through an additive stress decomposition,^[^ ^32^ ^]^ given by:

(1) $$T=\underset{\text{Passive}}{\underset{︸}{\frac{1}{J}\bar{F}\frac{\partial \;W^{dev}}{\partial \bar{E}}{\bar{F}}^{T}+\frac{\partial \;W^{vol}}{\partial \;J}}}+\underset{\text{Active}}{\underset{︸}{\frac{1}{J}FS^{act}F^{T}}}$$

where **T** represents the total Cauchy stress, **F** is the deformation gradient, *J* denotes volumetric changes in deformation, and $\bar{F}=J^{-\frac{1}{3}}\;F$ represents the deviatoric part of **F**. The passive stress follows a Fung‐type strain energy function *W*(**E**, *J*) given by Ref. [33]:

(2) $$W\left(E,J\right)=\underset{W^{dev}}{\underset{︸}{c\left[\exp(Q(E))-1\right]}}+\underset{W^{vol}}{\underset{︸}{\frac{K}{2}\left(\frac{J^{2}-1}{2}-\ln\left(J\right)\right)}}$$

where *W* ^*dev*^ and *W* ^*vol*^ denote the deviatoric and volumetric components of *W*, and $\bar{E}=\frac{1}{2}\left({\bar{F}}^{T}\bar{F}-I\right)$ is the Green‐Lagrange strain tensor. The quadratic form *Q* is expressed as: $Q=B_{1}{\bar{E}}_{11}^{2}+B_{2}\left({\bar{E}}_{22}^{2}+{\bar{E}}_{33}^{2}+{\bar{E}}_{23}^{2}\right)+B_{3}\left({\bar{E}}_{12}^{2}+{\bar{E}}_{13}^{2}\right)$ relative to the Cartesian coordinate system {**e**_1_, **e**_2_, **e**_3_}, with **e**_1_ indicating the local fiber direction. Constants *B*_1_, *B*_2_, and *B*_3_ are dimensionless constants representing local anisotropy in the myocardium, *c* is a positive stress‐like constant, and *K* is the bulk modulus.

A constitutive equation for the active stress can be written in terms of the second Piola–Kirchhoff active stress tensor as

(3) $$S^{act}=\frac{T_{a}\left(E_{f}\right)}{2E_{f}+1}\;N⊗N$$

where *T*_*a*_(*E*_*f*_) is a stress‐like positive function of the strain in the fiber direction **N** given by *E*_*f*_ = **N** · **E** **N**. The following form for *T*_*a*_(*E*_*f*_) was chosen

(4) $$T_{a}\left(E_{f}\right)=T_{{\mathrm{Ca}}^{2+}}\left[1+\beta \left(\sqrt{2E_{f}+1}-1\right)\right]$$

following the Hunter‐McCulloch‐TerKeurs model^[^ ^34^ ^]^ for the mechanical behavior of a contractile myocyte.

The passive behavior of the myocardium was characterized by four material parameters. Specifically, distinct material parameters were assigned to healthy and infarcted regions. For the healthy remote myocardium, the parameters $c_{r},B_{1}^{r},B_{2}^{r},B_{3}^{r}$ were utilized, while the infarct region employed parameters $c_{i},B_{1}^{i},B_{2}^{i},B_{3}^{i}$. These parameter values were determined through an analytical fitting process of the constitutive model (Equation (1)) to biaxial experimental data.^[^ ^31^ ^]^ The active force parameter $T_{{\mathrm{Ca}}^{2+}}$ was estimated by reproducing the experimental P‐V loop and minimizing any differences between the simulated and experimental results. The infarct region had no contractile behavior. To conduct forward FE simulations, both passive and active properties were assigned to these geometries. Detailed information regarding the experimental measurements and the corresponding properties obtained from those measurements is described by Mendiola et al.^[^ ^30^ ^]^

#### 2.2.4 Generation of RCCM Library

A comprehensive library comprising 592 diverse heart models was developed, building upon the initial four models. Random infarct regions were created in the LV in all the reconstructed hearts as described below (Figure 1D). Statistical analysis was conducted to elucidate the correlation between the average strains of infarcts and both the percentage of infarct size in the LV, as well as the stiffness of the LV (Figure S1, Supporting Information). A random point in LV was selected, serving as the starting location for each infarct. Next, a random percentage of the total elements, ranging from 5% to 60%, was chosen to determine the infarct size at that particular location. This process was repeated for all the reconstructed hearts, totaling 592 examples, generating a diverse set of heart models with infarcts of varying sizes and locations across the LV. To ensure a non‐repetitive and evenly distributed selection of infarct locations and sizes, Latin hypercube sampling (LHS) methodology was employed, which enabled systematic and uniform coverage of the parameter space, thus mitigating any potential bias in the infarct generation process. Inverse optimization was used to determine stiffness values in the infarct regions using biaxial stress–strain data for all post‐MI timepoints (n = 6 per timepoint).^[^ ^30^, ^31^ ^]^ The standard deviations of the stiffness values did not exceed 30% (relative to the mean values) for any post‐MI timepoint. Therefore, a LHS approach was applied with 30% upper and lower bounds (relative to the mean value) to generate the stiffness values. This way, distinct stiffness values were generated for each example. A forward simulation was performed with each unique cardiac model in the library, simulating a full cardiac cycle. Following the forward simulation, cardiac CRL strains were obtained for the entire heart at ES (ES; Figure 1).

### 2.3 Preprocessing of ML Inputs

#### 2.3.1 American Heart Association‐Based Representation of Strains

**Figure 2:**
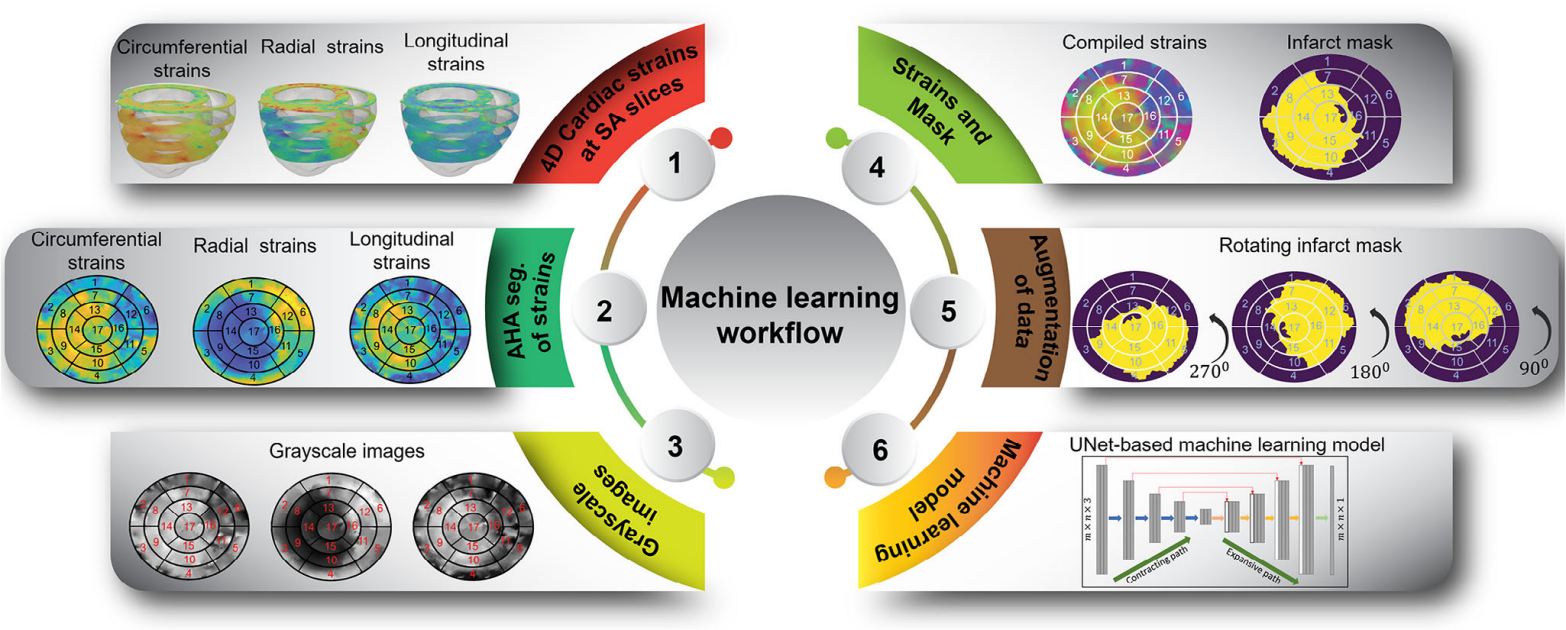
Preprocessing steps: The cardiac circumferential, radial, and longitudinal (CRL) strains of the left ventricle (LV) were acquired at the base, mid, apical, and apex levels. These strain values were then mapped onto standard American Heart Association (AHA) bullseye maps and subsequently transformed into greyscale images. The integration of these three sets of images, together with their corresponding infarct masks, constituted the dataset employed for machine learning (ML) training after the application of data augmentation techniques.

The focus of this study was to locate infarcts within the LV. For this purpose, the cardiac CRL strains of LV were obtained at the base, mid, apical, and apex short‐axes, representing four common short‐axis sections in routine CMR scans. Next, the strain data was translated into standard American Heart Association (AHA) bullseye maps (**Figure** **2**) showcasing the spatial distribution of the strains across the LV. This conversion procedure was executed for each CRL strain, thereby generating three distinct AHA‐based bullseye maps per simulation.^[^ ^35^ ^]^ The strain maps were stored in images to facilitate further analysis and streamline the input features for ML algorithms. This image‐based representation ensured consistency in the input features and allowed for easy preprocessing prior to feeding images into the ML models. By adopting this approach, the strains were effectively visualized in terms of pixels, enabling effective data preprocessing techniques and smooth and cohesive integration with ML models.

#### 2.3.2 Conversion to Greyscale Images and Normalization

The bullseye maps representing CRL strains served as preliminary inputs to our ML models (Figure 2). Appropriate preprocessing steps were deemed necessary to optimize the efficiency of using three separate bullseye maps as inputs. In particular, recognizing that the bullseye maps could be generated using any color scheme while maintaining consistent color intensity throughout, a transformation was performed to convert the RGB images representing each type of CRL strain into greyscale images, effectively transitioning from three‐channel images to one‐channel images. Subsequently, these greyscale CRL images were combined into a single RGB image, wherein the red (R), green (G), and blue (B) channels represented the greyscale image of circumferential, radial, and longitudinal strains, respectively. Having unified the greyscale CRL strain images into a multi‐channel representation, an essential normalization step was subsequently performed. This normalization process standardized the pixel values across the entire image, thereby promoting smoother convergence during the training of the ML models. By applying this series of preprocessing steps, the objective was to enhance the efficacy of our ML algorithms. The transition to greyscale images and pixel normalization not only accelerated training convergence but also facilitated more meaningful and efficient utilization of the bullseye maps as input features for our ML models. Ultimately, these preprocessing measures played a pivotal role in enhancing the overall performance and accuracy of the ML‐driven CRL strain prediction framework.

#### 2.3.3 Data Augmentation

After the necessary preprocessing steps, the methodology involves data augmentation to enhance the diversity and robustness of the training process. To this end, each channel of the image was rotated by 90, 180, and 270 degrees as part of a rotation augmentation procedure. This process resulted in a fourfold increase in the number of images within the training dataset. Next, the augmented dataset was partitioned into distinct sets for training and validation purposes, facilitating the training of the ML model. A detailed breakdown of the exact number of images at each stage of the data splitting and augmentation process is presented in Figure 5. Using the fixed rotation strategy instead of random rotation, the misalignment of the images was avoided and consistency across the dataset was ensured. In addition, the rotation procedure was applied after resizing the images into equal width and height for further consistency. This procedure was expected to increase the adaptability of the model by enhancing its capacity for generalization.

### 2.4 Unet‐Based Multi‐Fidelity Models

**Figure 3:**
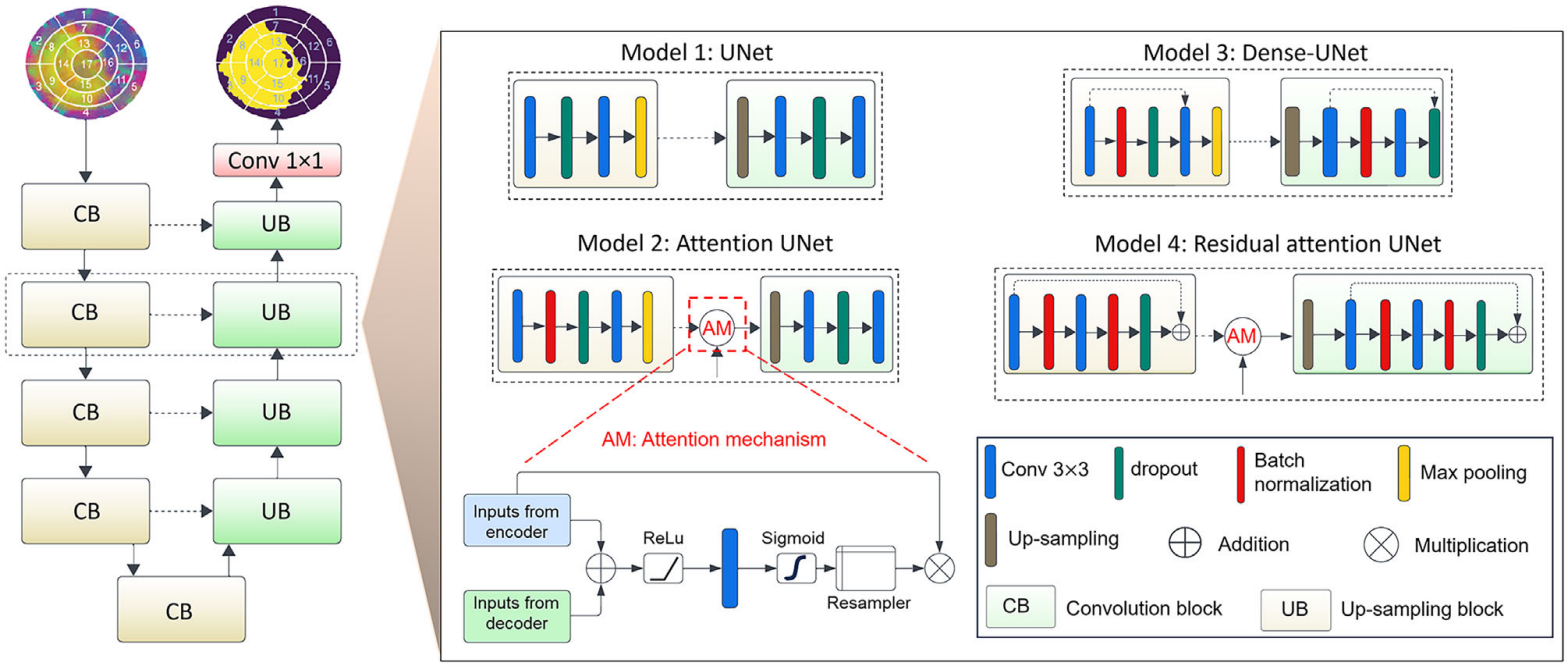
Illustration of base UNet and its variants for infarct segmentation in LV strain images. The base UNet architecture features a typical encoder‐decoder structure. Attention UNet incorporates an attention mechanism in the encoder layers to focus on relevant features. Dense UNet adopts dense connections in the encoder to facilitate information flow. Residual attention UNet combines both residual connections and attention mechanisms for improved feature extraction and information preservation.

The aim was to develop and apply a multi‐fidelity ML model to identify infarct regions from LV strain data represented as three‐channel images, where each channel corresponds to a distinct directional strain. The multi‐fidelity model takes advantage of extensive rodent‐based training data (referred to as low‐fidelity) while improving the predictions by using limited human training data (referred to as high‐fidelity). The performance of multiple UNet models (**Figure** **3**), which are described in detail in Section S2 (Supporting Information), was compared in identifying infarct regions from low‐fidelity data, i.e., RCCM LV strain data. Further specifics, including operation type, resolution size, and channel count, are provided in **Table** **1**. After identifying the ML model with the highest performance among the low‐fidelity‐trained UNet models, its performance was subsequently tested using high‐fidelity human CMR data described in Section 2.6, which underwent a series of preparatory steps delineated in Section 2.3 to predict infarct regions in human patients.

**Figure 4:**
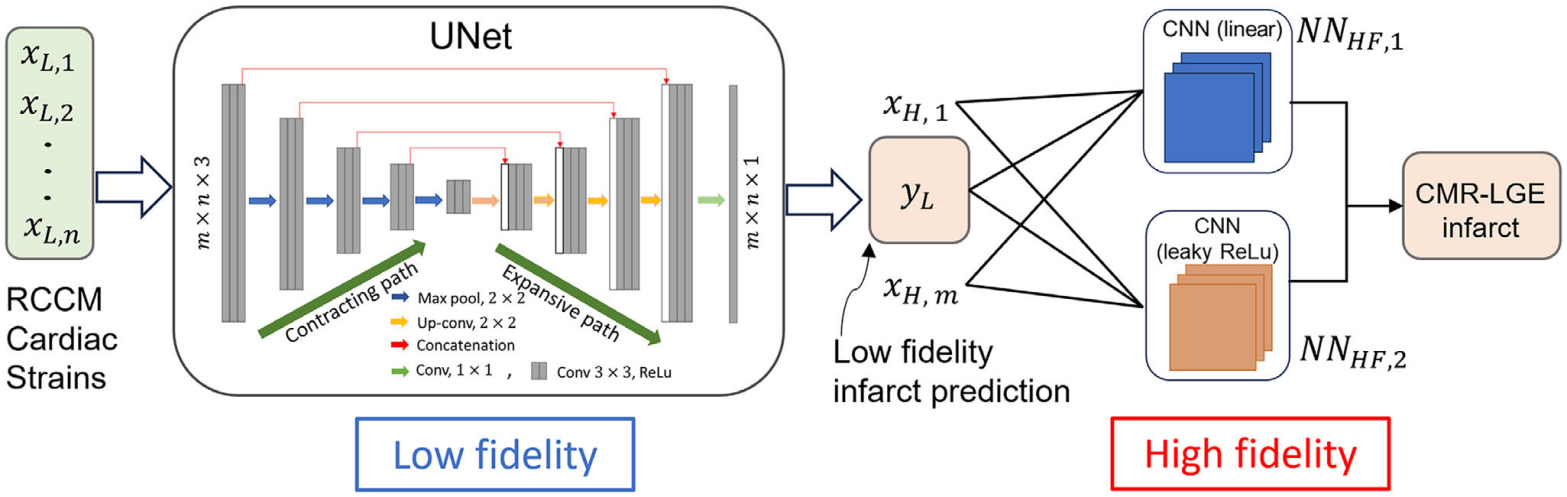
Network architecture highlighting the composite neural network that learns from the multi‐fidelity data. The first block (UNet) constitutes the low‐fidelity deep neural network *NN*_*LF*_(*x*_*L*_, θ), followed by two high‐fidelity models including *NN*_*HF*1_(*x*_*H*_, *y*_*L*_, γ_1_) and *NN*_*HF*2_(*x*_*H*_, *y*_*L*_, γ_2_), consisting of CNN kernels without and with leaky ReLU activation functions, respectively. The combined output of the two high‐fidelity neural networks is used to predict the binary infarct mask *y*_*H*_.

**Table 1:** Detailed architecture of UNet, emphasizing the contracting and expansive paths, including block types, output resolutions, and channel numbers at different stages. The final output layer generates a single‐channel image with a resolution the same as the input image.

Contracting path			Expansive path		
Block type	Output resolution	Output channel number	Block type	Output resolution	Output channel number
Input	128 × 128	3	Output	128 × 128	1
2 blocks Conv 3 × 3	128 × 128	16	Conv 1 × 1	128 × 128	1
Max pooling			2 blocks Conv 3 × 3	128 × 128	16
2 blocks Conv 3 × 3	64 × 64	32	Up‐conv Concatenate	128 × 128	16
Max pooling			2 blocks Conv 3 × 3	64 × 64	32
2 blocks Conv 3 × 3	32 × 32	64	Up‐conv Concatenate	64 × 64	32
Max pooling			2 blocks Conv 3 × 3	32 × 32	64
2 blocks Conv 3 × 3	16 × 16	128	Up‐Conv Concatenate	32 × 32	64
Max pooling			2 blocks Conv 3 × 3	16 × 16	128
2 blocks Conv 3 × 3	8 × 8	256	Up‐Conv Concatenate	16 × 16	128

To ensure that the model effectively reconciled low and high‐fidelity differences and maintained generalizability across species, the strategies described in the following sections were employed.

#### 2.4.1 Hierarchical Multi‐Network Architecture for Adaptation

The multi‐fidelity approach was built on a composite neural network architecture to reconcile the differences between low‐fidelity and high‐fidelity data.^[^ ^36^ ^]^ This architecture consisted of three interconnected networks: *NN*_*LF*_, *NN*_*HF*1_, and *NN*_*HF*2_. First, the *NN*_*LF*_ (low‐fidelity network), based on a UNet architecture, was trained on extensive rodent data. *NN*_*LF*_ effectively learns generalized infarct patterns and captures essential spatial features of myocardial infarcts derived from computationally simulated strains. However, while *NN*_*LF*_ provides a strong foundational understanding, it did not fully represent the specific nuances of the human myocardium. To address this, the *NN*_*HF*1_ (high‐fidelity network 1) applied a linear transformation to the output of *NN*_*LF*_, aligning it with the human data scale. This linear adjustment accounts for variations in cardiac geometry and strain amplitudes between species, ensuring the model's output matches the scale of high‐fidelity data.

Next, the *NN*_*HF*2_ (high‐fidelity network 2) introduced non‐linear corrections using a convolutional kernel with leaky ReLU activation. *NN*_*HF*2_ further refines the predictions to adjust complex physiological discrepancies between rodents and humans, such as species‐specific strain responses and variations in myocardial mechanics. The combined output from *NN*_*LF*_, *NN*_*HF*1_, and *NN*_*HF*2_ provides an infarct mask that was better aligned with human CMR data, ensuring high‐fidelity prediction accuracy enhances the model's generalizability across species.

#### 2.4.2 Generalized Regressive Scheme Approach

A generalized regressive scheme^[^ ^22^ ^]^ was employed to map low‐fidelity rodent data onto high‐fidelity human outputs, ensuring that the model accurately captures both linear and non‐linear correlations between the two data types. This scheme is mathematically expressed as

(5) $$y_{H}=f_{l}\left(x_{H},y_{L}\right)+f_{\mathrm{nl}}\left(x_{H},y_{L}\right)$$

here f_*l*_ and f_*nl*_ represent linear and nonlinear functions, respectively, to map the low‐fidelity data to the high‐fidelity data, learned by the neural networks, *NN*_*HF*1_ and *NN*_*HF*2_. Here, *y*_*L*_ is low fidelity output (infarct mask), *x*_*H*_ denotes high fidelity inputs (strains), and *y*_*H*_ is high fidelity output (infarct mask). The network architecture of the multi‐fidelity deep neural network is composed of three neural networks, shown in **Figure** **4**. The first one, *NN*_*LF*_(*x*_*L*_, θ), is UNet, which is the same as previously discussed UNet in Section S2 (Supporting Information), while the second NN, *NN*_*HF*1_(*x*_*H*_, *y*_*L*_, γ_1_), is CNN kernel with 10x10 filter and third NN, *NN*_*HF*2_(*x*_*H*_, *y*_*L*_, γ_2_), is also CNN kernel of same size but with leaky ReLU activation. Here, θ, γ_1_, γ_2_ indicates weights of *NN*_*LF*_, *NN*_*HF*1_ and *NN*_*HF*2_, respectively.

#### 2.4.3 Model Generalizability and Cross‐Validation

To assess the generalizability of the model with limited high‐fidelity human data (total *n* = 10, *n* = 5 in the main experiment), data augmentation was implemented by rotating the strain maps of each patient by 90°, 180°, and 270°, increasing the training set size to 16 samples, as detailed in Section 2.3. The model was first trained using strain data from two patients mixed with low‐fidelity rodent data and tested it on the remaining three, repeating this procedure until all patients were independently tested. To further investigate the effect of incorporating more high‐fidelity information, the process was repeated by training with four patients and testing on the remaining one, rotating the held‐out subject in each iteration. These experiments enabled to evaluate how performance improved as the number of human samples in training increased. Additionally, to more comprehensively assess the model's generalizability, a final leave‐one‐out cross‐validation (LOOCV) experiment was performed on an expanded cohort of ten human patients, which included both ischemic and non‐ischemic cases (Section S3, Supporting Information). In this setup, nine patients were used for training and one was held out for testing, repeated across all subjects. This approach reinforced the model's ability to generalize across different human subjects and validated its reliability for real‐world applications.

### 2.5 Ml Training Process and Optimization

As the attention UNet, dense UNet, and residual attention UNet were drawn from the UNet, the UNet ML model was utilized to explore general hyperparameters for identifying infarct regions. This exploration included carefully selecting input image dimensions, specifically 512x512, 256x256, and 128x128. A variety of loss functions, including binary cross‐entropy (BCE) loss, dice similarity coefficient (DSC) loss, and the intersection over union (IoU) loss, were investigated to effectively minimize the dissimilarity between the predicted infarct regions and the corresponding ground‐truth mask during training. The model performance evaluation was based on accuracy, precision, recall, IoU, and DSC evaluation metrics. The model was optimized using a gradient‐based optimization algorithm (Adam) to minimize the chosen loss function. The following briefly describes the loss functions and evaluation metrics used in this work.

**Figure 5:**
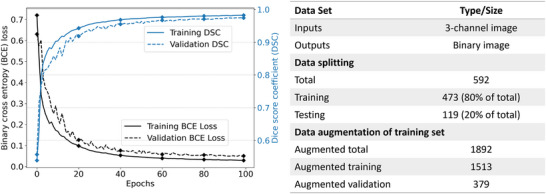
Training machine learning model using different loss functions including binary cross entropy (BCE), dice similarity coefficient (DSC) and the intersection over union (IoU) loss functions. 100 epochs, patience is five, batch size is 128, image size is 128 by 128, and all images were normalized before training.

#### 2.5.1 Loss Functions

The BCE loss was a commonly used loss function for binary pixel‐wise segmentation tasks.^[^ ^37^ ^]^ It measures the pixel‐wise dissimilarity between the predicted segmentation map (*p*) and the corresponding ground‐truth mask (*y*). The BCE loss is defined as:

(6) $$BCE\left(p,y\right)=-\frac{1}{N}\sum_{i=1}^{N}\left[y_{i}.log\left(p_{i}\right)+\left(1-y_{i}\right).log\left(1-p_{i}\right)\right]$$

where *N* represents the total number of pixels in the segmentation map. The BCE loss penalizes incorrect predictions while encouraging the model to produce probabilistic values close to one for pixels in infarct regions and close to zero for pixels in healthy regions.

The DSC loss is an additional loss function to enhance spatial overlap between the two masks. The DSC loss is defined as:

(7) $$DSC\;loss=1-\frac{2.\sum_{i}p_{i}.y_{i}}{\sum_{i}p_{i}+\sum_{i}y_{i}}$$

Similar to the DSC loss function, the intersection over union (IoU) was another commonly used loss function for segmentation. It also measured the spatial overlap between the predicted segmentation map (*p*) and the ground‐truth mask (*y*). The IoU loss is given by:

(8) $$IoU\left(p,y\right)=1-\frac{\sum_{i}p_{i}\cdot y_{i}}{\sum_{i}\left(p_{i}+y_{i}-p_{i}\cdot y_{i}\right)}$$

By optimizing the DSC and IoU loss functions, the model learns to produce precise segmentation maps that accurately delineate infarct regions. In the above equations, *p*_*i*_ represents the pixel value in the predicted map at *i*th pixel, and *y*_*i*_ was the pixel value in the ground‐truth mask at *i*th pixel. The summation spans all pixels in the image. A smaller loss function value indicates improved segmentation accuracy, signifying greater overlap between the predicted and true masks.

The trainable variables for the multi‐fidelity neural network were learned by minimizing the following loss function which was minimized using the Adam optimizer:

(9) $$MSE=MSE_{L}+MSE_{H}+\lambda \Sigma \beta_{i}^{2}$$

where

(10) $$MSE_{L}=\frac{1}{N_{L}}\Sigma_{i=1}^{N_{L}}\left(|p_{i_{L}}-y_{i_{L}}{|}^{2}\right)$$

(11) $$MSE_{H}=\frac{1}{N_{H}}\Sigma_{i=1}^{N_{H}}\left(|p_{i_{H}}-y_{i_{H}}{|}^{2}\right)$$

here, *N*_*L*_ indicates the total number of training samples in low‐fidelity dataset, and *N*_*H*_ indicates the total number of training samples in the high‐fidelity dataset. $p_{i_{L}}$ and $p_{i_{H}}$ denotes the predicted outputs of the *NN*_*L*_ and *NN*_*H*_, β is any weight in *NN*_*L*_ and *NN*_*H*_ and λ is the L2 regularization rate for β.

#### 2.5.2 Evaluation Metrics

Several well‐known metrics, including accuracy, precision, and recall,^[^ ^38^ ^]^ were used to evaluate models' performance to segment infarct regions, which provided important insights into the models' overall effectiveness for prediction. However, two additional metrics, namely IoU and DSC, hold particular significance for infarct region segmentation tasks, where the spatial overlap was critical. These metrics, described below, were crucial for assessing how well the model could locate the infarct regions within the LV myocardium.

The IoU, also called the Jaccard index, quantifies the extent of the region where the ground‐truth mask and predicted segmentation map overlap. It was determined by dividing the intersection of the predicted and actual masks by their union:

(12) $$IoU=\frac{\left|P\cap G\right|}{\left|P|+|G|-|P\cap G\right|}$$

where |*P*| is the predicted pixels, |*G*| is pixels of ground truth and |*P*∩*G*| represents the number of pixels in the intersection of the predicted and ground‐truth. The predicted and ground‐truth masks must perfectly overlap for the IoU to be 1, which ranges from 0 to 1. Increased segmentation accuracy was shown by a higher IoU score, which measured the degree of spatial overlap between the model's predictions and the actual infarct regions.

The DSC, also referred to as the F1‐score, was another metric that measures the spatial overlap between the predicted infarct region with the ground‐truth infarct region. It was defined as twice the intersection of the predicted and ground‐truth masks divided by their sum:

(13) $$DSC=\frac{2\cdot |P\cap G|}{\left|P|+|G\right|}$$

Similar to the IoU, the DSC ranges from 0 to 1, with 1 representing a perfect segmentation. DSC was particularly useful in evaluating the segmentation accuracy of smaller infarct regions, as it considered the relative contribution of true positive, false positive, and false negative predictions. To ensure consistency and anatomical relevance, all evaluation metrics were computed exclusively within a circular region of interest, corresponding to the LV myocardium. This circular mask excluded the white corners of the image, which were not part of the AHA segmentation. Importantly, the DSC was calculated solely on the pathological class (i.e., infarcted pixels), and background or non‐infarct areas were not included in the metric (Figure S4, Supporting Information). This ensured that the DSC reflected only the spatial overlap of infarcted regions without any inflation from non‐pathological background agreement (Section S4, Supporting Information).

### 2.6 Human LGE‐CMR Used for Validation

Human LGE‐CMR data was collected from ten patients (*n* = 10) with confirmed MI. All personal identifiers were removed in accordance with the National Institute of Health de‐identification protocol, comprising 18 elements considered to be protected health information (https://privacyruleandresearch.nih.gov/pr_08.asp).

#### 2.6.1 CMR Image Acquisition

To validate the trained single and multi‐fidelity models, cine CMR scans were acquired using a 3.0‐T clinical scanner (Siemens Verio; Siemens, Erlangen, Germany) with phased‐array coil systems. CMR scans included a short axis stack using a steady‐state free‐precession sequence. LGE images were acquired at slice positions matched to cine CMR scans approximately 10 to 15 min after intravenous gadolinium‐based contrast administration (gadopentetate dimeglumine, gadoterate meglumine; 0.15 mmolkg^−1^) with in‐plane spatial resolutions of 1.8 mm by 1.3 mm and slice thicknesses of 6 to 7 mm with 3 to 4 mm gap. Additional details of LGE‐CMR scans are available in Ref. [39, 40].

#### 2.6.2 Strain Calculation Using Image Registration

The CRL strains from cine CMR slices were calculated at ES, using the ED frame as a reference point. To achieve this, an optimization‐based image registration approach^[^ ^41^, ^42^, ^43^ ^]^ was implemented. The registration determines the optimal transformation that aligns a reference frame, denoted by F, with a moving image, denoted by M. This alignment was achieved by minimizing the cost function, denoted by Ψ, as expressed through the following equation:

(14) $$\Psi \left(c,u\right)=\frac{1}{\sigma_{i}^{2}}{\left\|F-M\circ c\right\|}^{2}+\frac{1}{\sigma_{x}^{2}}{\left\|u-c\right\|}^{2}+\frac{1}{\sigma_{T}^{2}}{\left\|\nabla u\right\|}^{2}$$

where σ_i_ and σ_x_ represent the noise intensities, while σ_T_ serves as a regularization factor. The term **u** denotes the parametric transformation, representing the Cartesian displacement vector at each pixel between two consecutive time frames. Simultaneously, **c** signifies the corresponding non‐parametric spatial transformation.

The large deformation theory was employed to derive the Green‐Lagrange strain tensor (**E**) for Cartesian displacements obtained from imaging as pointwise data. The strains were computed utilizing the total deformation gradient (**F**) and the identity matrix (**I**) in accordance with the expression: $E=\frac{1}{2}\left(F^{T}F-I\right)$. Here, **F** was determined as the propagation of the deformation gradient between two consecutive load increments (**F_i_**), given by:

(15) $$F=\prod_{i}F_{i};F_{i}=I+\frac{\partial u}{\partial X}\;=\left[\begin{array}{ccc} \frac{\partial u_{x}}{\partial X} & \;\frac{\partial u_{x}}{\partial Y} & \;\frac{\partial u_{x}}{\partial Z}\\ \frac{\partial u_{y}}{\partial X} & \;\frac{\partial u_{y}}{\partial Y} & \;\frac{\partial u_{y}}{\partial Z}\\ \frac{\partial u_{z}}{\partial X} & \;\frac{\partial u_{z}}{\partial Y} & \;\frac{\partial u_{z}}{\partial Z} \end{array}\right].$$

here, u_x_, u_y_, and u_z_ denote the displacements in the x, y, and z directions between two consecutive load increments. Finally, CRL strains were obtained by transforming the Cartesian strains into CRL axes using an orthonormal transformation matrix (**Q**) as:

(16) $$E_{\left[C,R,L\right]}={\mathrm{QEQ}}^{T}\;=\left[\begin{array}{ccc} E_{\mathrm{CC}} & \;E_{\mathrm{CR}} & \;E_{\mathrm{CL}}\\ E_{\mathrm{RC}} & \;E_{\mathrm{RR}} & \;E_{\mathrm{RL}}\\ E_{\mathrm{LC}} & \;E_{\mathrm{LR}} & \;E_{\mathrm{LL}} \end{array}\right]$$

where *E*_*CC*_, *E*_*RR*_, and *E*_*LL*_ represent the circumferential, radial, and longitudinal strains, respectively. The strains calculated from CMR scans and preprocessed, as described in Section 2.3, were used as inputs to the models to predict MI regions. ML models trained on only RCCM data (single‐fidelity) were first evaluated by predicting infarct regions of all five human patients. For multi‐fidelity training, two human strain datasets were initially combined with low‐fidelity data to train the model, which was then tested on the remaining three patients. This process was repeated across patient combinations to assess prediction consistency. To investigate the impact of additional high‐fidelity data, the model was next trained using four human patients and tested on the fifth, rotating the held‐out subject. These experiments provided insight into how the inclusion of more human data improved prediction accuracy. A final LOOCV using an expanded ten‐patient cohort was also performed to more comprehensively evaluate generalizability across diverse scar pathologies, including ischemic and non‐ischemic patients, as detailed in Section S3 (Supporting Information). The variation in the number of human patients in the training set was used to evaluate the influence of human data inclusion on the performance of the multi‐fidelity model.

## 3 Results

A comprehensive analysis of the predictive capability of the encoder‐decoder‐based ML models for infarct region identification from LV strain was conducted. We began with an extensive examination of different image sizes in the ML model to determine the optimal image size for training to enhance the ability of the model to capture fine‐grained features. This comparison was carried out under the same experimental conditions, considering the uniform data preprocessing and utilization of the same loss function. Next, we investigated the impact of various loss functions on the performance of the ML model while keeping the image size fixed. These loss functions, including the BCE, DSC, and IoU loss, were evaluated using carefully chosen evaluation metrics. Furthermore, the efficacy of all chosen encoder‐decoder‐based architectures was compared against each other. We selected the best‐performing architecture as single‐fidelity NN to further extend the framework to multi‐fidelity NN. Finally, we tested the multi‐fidelity trained NN in predicting the infarct region from human LGE‐CMR‐derived strains. This evaluation aimed to demonstrate the model's effectiveness when applied to data from human patients, thereby evaluating its capacity for generalization and its suitability for clinical application.

### 3.1 Influence of Image Size on Model Adaptability

We considered three distinct image sizes for experimentation: 128x128, 256x256, and 512x512 pixels. Each image size was preprocessed using the steps detailed in the Methods section, and the BCE loss function was employed consistently to ensure fair and unbiased comparisons. The results of this investigation are presented in **Table** **2**a, where the performance metrics, including accuracy, precision, recall, IoU, and DSC scores, are listed for each image size. Our analysis revealed a remarkable consistency in model performance across varying image sizes. The IoU and DSC metrics exhibited minimal differences, underscoring the model's adaptability across image sizes. Notably, even the 128x128 image size, which is often associated with reduced spatial information, provided IoU (0.9510) and DSC (0.9744) values indicative of effective infarct region localization. The 256x256 and 512x512 image sizes also showcased comparable spatial overlap and segmentation accuracy results. Given this consistency in performance, it was deemed justifiable to make use of the 128x128 picture size for later training stages. Using a reduced image size reduced computation and training time while maintaining high accuracy.

**Table 2:** Comparison of different image sizes and loss functions in UNet ML models.

(a) Different image sizes in UNet					
Image sizes	Accuracy	Precision	Recall	IoU	DSC
128 × 128	0.9836	0.9854	0.9646	0.9510	0.9744
256 × 256	0.9846	0.9880	0.9619	0.9506	0.9743
512 × 512	0.9844	0.9867	0.9616	0.9491	0.9735

(b) Different loss functions in UNet					
Loss functions	Accuracy	Precision	Recall	IoU	DSC
BCE loss	0.9814	0.9697	0.9752	0.9461	0.9720
IoU loss	0.9759	0.9597	0.9693	0.9312	0.9640
DSC loss	0.9795	0.9687	0.9698	0.9402	0.9688

### 3.2 Quantitative Analysis of Loss Functions

**Figure 6:**
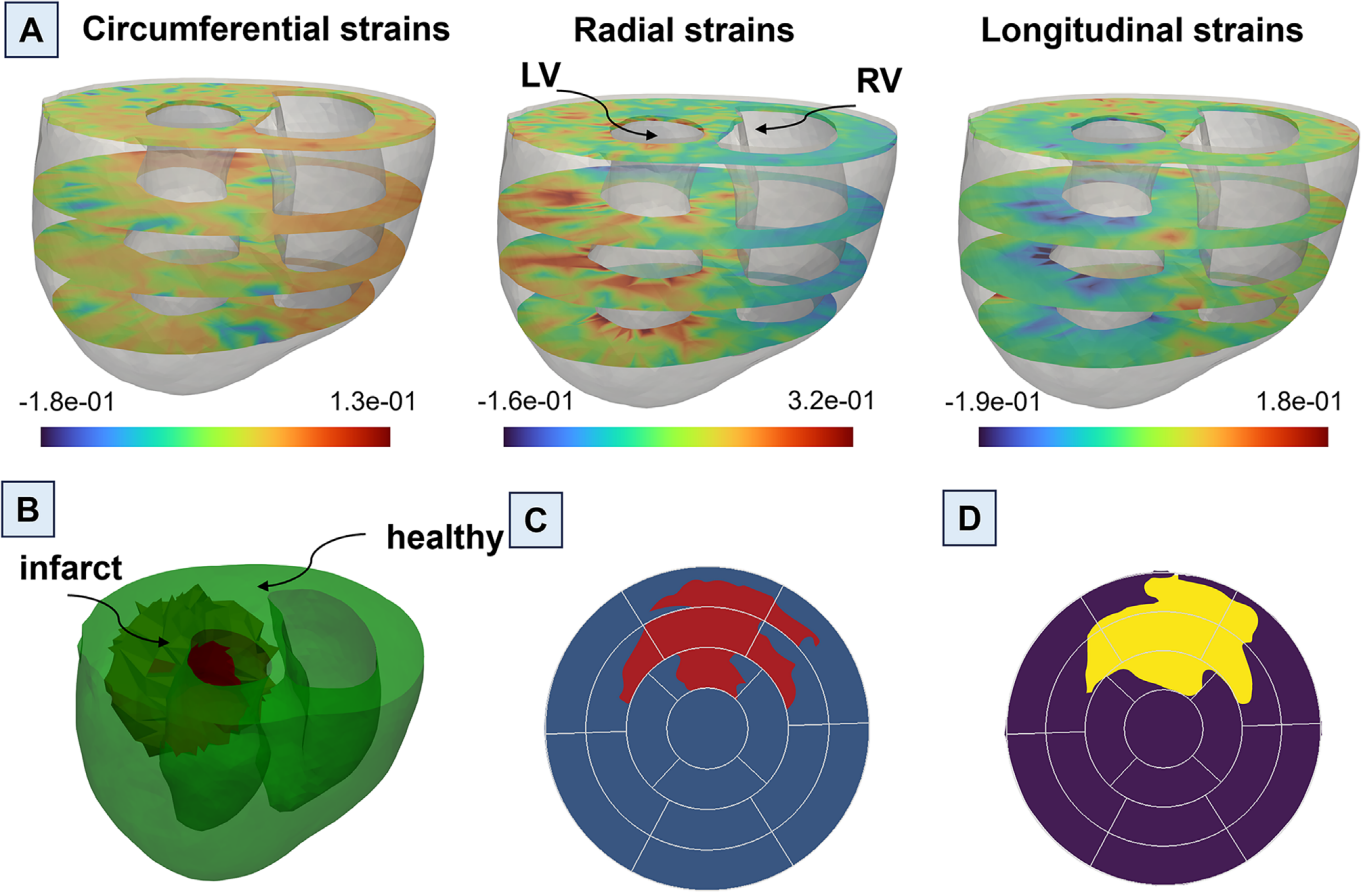
A representative example showcasing the prediction of the infarct region using the UNet machine learning (ML) model based on cardiac circumferential, radial, and longitudinal (CRL) strains obtained from finite element (FE) simulation. A) Cardiac CRL strains depicted at the base, mid, apical, and apex of the left ventricle. B) A 3D image of the heart model, where healthy regions are represented in green and the infarct region in red. C) An American Heart Association (AHA) bullseye map derived from the 3D heart model representation, indicating healthy (blue) and infarct (red) regions. D) ML‐predicted healthy (purple) and infarct (yellow) in the AHA bullseye map format.

**Figure 7:**
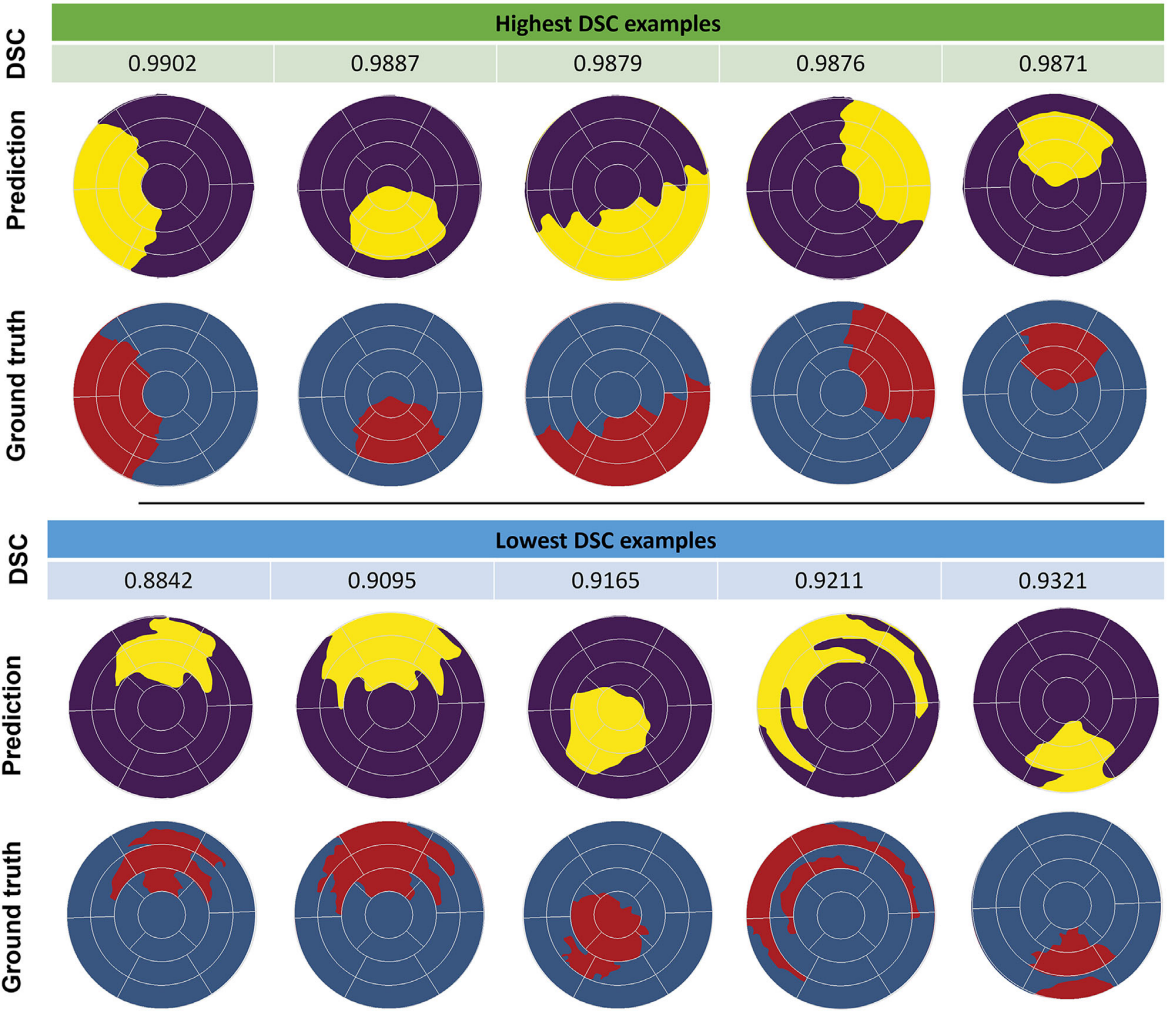
Visual comparison of single‐fidelity finite element (FE) ground truth against UNet predictions, showcasing the highest and lowest dice similarity coefficient (DSC) scores. Five examples each highlight the model's exceptional accuracy in infarct region delineation, revealing both optimal and challenging predictions.

The effectiveness of several loss functions, namely BCE, DSC, and IoU loss, was examined in UNet. The quantitative analysis was performed by comparison of metric scores, including accuracy, precision, recall, IoU, and DSC for each loss function (Table 2b). All loss functions exhibited comparable accuracy. Specifically, BCE obtained a DSC of 0.9720 and an IoU of 0.9461, whereas the IoU loss function displayed a DSC of 0.9640 and an IoU of 0.9312. The DSC loss function produced a DSC of 0.9688 and an IoU of 0.9402. For brevity, all ML predictions of the FE results shown in **Figures** **6** and **7** were based on the training using the BCE loss function. However, models trained with different loss functions were subsequently evaluated on human CMR‐derived strains (Section 3.4).

### 3.3 Impact of Different Architectures on Performance Metrics

All four distinct models, namely UNet, attention UNet, dense UNet, and residual attention UNet, required trainable parameters on the order of millions (**Table** **3**). UNet demonstrated a notable reduction in parameter count compared to other models, while all utilized models remained parameter‐efficient compared to other segmentation models.^[^ ^44^, ^45^, ^46^ ^]^ Although UNet streamlined its parameter count, its performance in infarct region identification remained exceptional. Evaluation metrics in **Table** **4** supported this observation, with UNet achieving an IoU of 0.9461 and DSC of 0.9720. Similarly, attention UNet, dense UNet, and residual attention UNet showed comparable metrics to UNet, although with slight variation in performance attributed to their distinct architectures. These results highlighted the balance between computational efficiency and segmentation accuracy in the UNet model. A representative example of the input data, ground truth infarct region, and UNet model prediction for one heart model is illustrated in Figure 6. Additionally, Figure 7 showcases a comparison between ground truth and prediction for examples with the highest and lowest DSC metric scores, demonstrating minimal variation between the predicted infarct regions and the ground truth masks even in examples with the lowest DSC scores. These findings emphasize the effectiveness of our approach, highlighting that the computationally less intensive UNet can achieve comparable high metrics when compared to attention UNet, dense UNet, and residual attention UNet architectures.

**Table 3:** Comparative analysis of trainable parameters in encoder‐decoder‐based ML architecture, illustrating the model complexity in relation to the increasing number of parameters.

ML models	Number of trainable parameters
UNet	1.94 million
Attention UNet	2.38 million
Dense UNet	2.70 million
Residual attention UNet	3.71 million

**Table 4:** Comprehensive evaluation of all the machine learning models across different metrics.

ML Model	Accuracy	Precision	Recall	IoU	DSC
UNet	0.9814	0.9697	0.9752	0.9461	0.9720
Attention UNet	0.9813	0.9765	0.9685	0.9462	0.9720
Dense UNet	0.9867	0.9844	0.9750	0.9601	0.9793
Residual attention UNet	0.9850	0.9784	0.9759	0.9552	0.9767

### 3.4 ML Model Performance Assessment with Human LGE‐CMR

**Figure 8:**
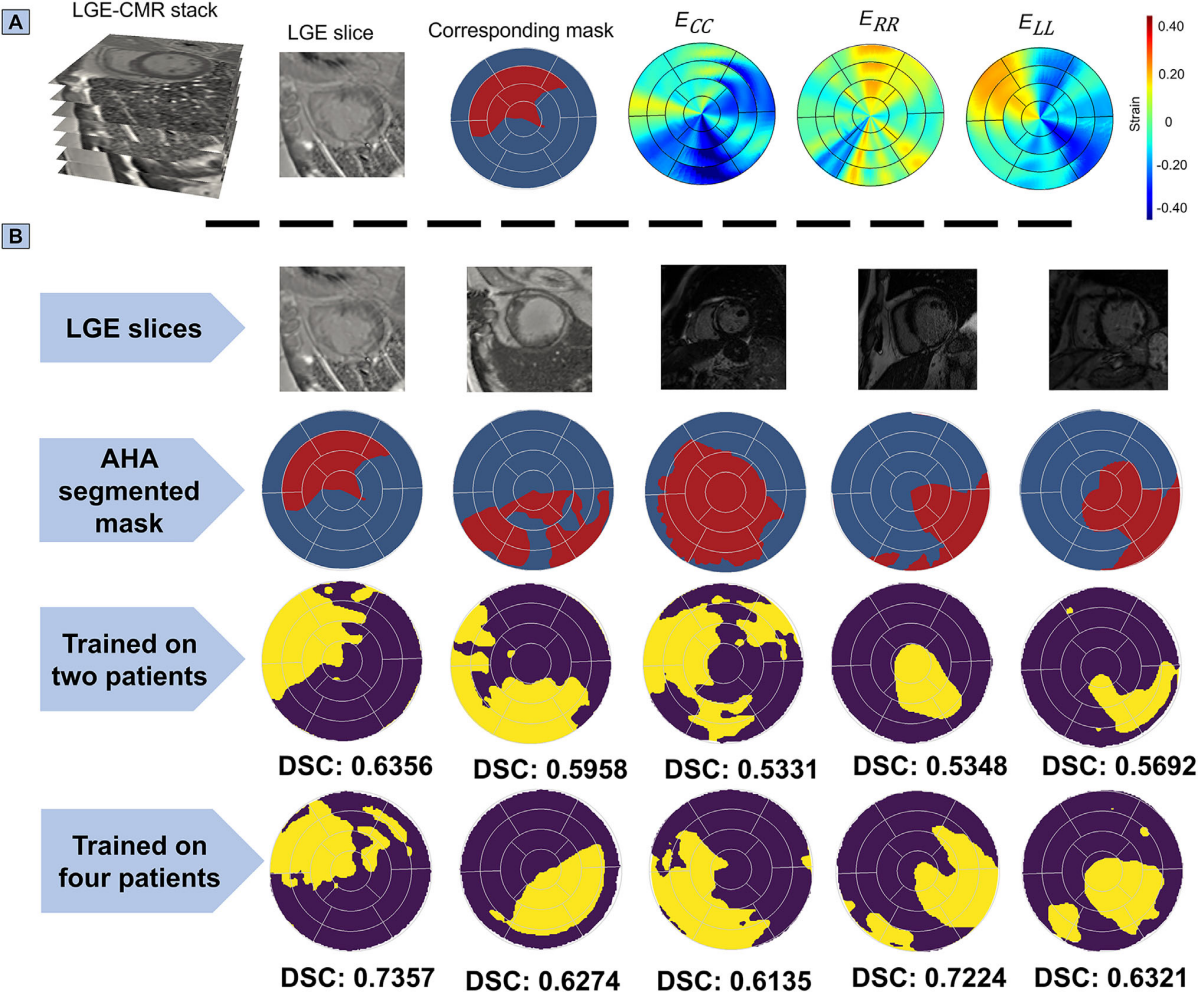
A) A stack of human late gadolinium‐enhanced cardiac magnetic resonance (LGE‐CMR) imaging slices with one of the infarcted LGE slices from the imaging stack to highlight the infarct and healthy regions on the American Heart Association (AHA) bullseye map. Cardiac circumferential, radial, and longitudinal (CRL) strains are also displayed for Patient #1 as a representative example. B) One of the infarcted LGE slices from the imaging stack is shown, with a corresponding AHA bullseye map indicating the healthy and infarct regions, which serve as the ground truth derived from LGE‐CMR. The Dice similarity coefficient (DSC) scores for the multi‐fidelity trained machine learning (ML) model were presented, showing results from models trained using both single‐fidelity and high‐fidelity human datasets. The model was tested on all human patients independently, with training performed on two patients simultaneously. Additionally, the DSC scores were also presented where the multi‐fidelity ML model was trained on four patients.

To rigorously evaluate our ML model performance and indicate the feasibility of the presented approach in the clinical setting, we used cardiac strain data from human LGE‐CMR scans that is accessible in clinical scenarios. By applying our trained ML model to the strain data calculated using image registration from CMR scans, we obtained predictions that were subsequently compared against the ground truth mask obtained from LGE‐CMR scans (**Figure** **8**). This comparison enabled a comprehensive evaluation of our model's predictive performance in identifying the precise infarct regions within the LV. The model's performance was evaluated using the DSC metric score (Figure 8) and other metrics detailed in **Table** **5**. In the initial evaluation using five human subjects, the DSC scores obtained from multi‐fidelity models trained with data from two human patients were 0.6356, 0.5958, 0.5331, 0.5348, and 0.5692 (referred to as Patient #1 through Patient #5), respectively (Figure 8). When the number of human patients in the training set was increased to four, the DSC scores improved to 0.7357, 0.6274, 0.6135, 0.7224, and 0.6321 for the same, respectively (Figure 8). These results demonstrated improved prediction accuracy for each patient when the model was trained with more high‐fidelity samples, highlighting the benefit of increasing the high‐fidelity dataset for enhanced model performance. A more comprehensive evaluation of model generalizability using an expanded ten‐patient cohort was presented in the supplementary material to complement these primary results (Section S3, Supporting Information). This extended cohort included patients with both ischemic and non‐ischemic myocardial scars, and the results suggested that the proposed model can effectively detect infarct regions across both scar types.

**Table 5:** Evaluation of multi‐fidelity models on human datasets trained with all single‐fidelity strain data and high‐fidelity strain data from two and four high‐fidelity human patients, with average metrics across all human patients.

ML Model	Accuracy	Precision	Recall	IoU	DSC
UNet trained on two human patients	0.7066	0.5769	0.5975	0.4033	0.5737
UNet trained on four human patients	0.7552	0.6898	0.6582	0.5010	0.6662

### 3.5 Effects of High‐Fidelity Sample Size on Multi‐Fidelity Model Prediction

**Figure 9:**
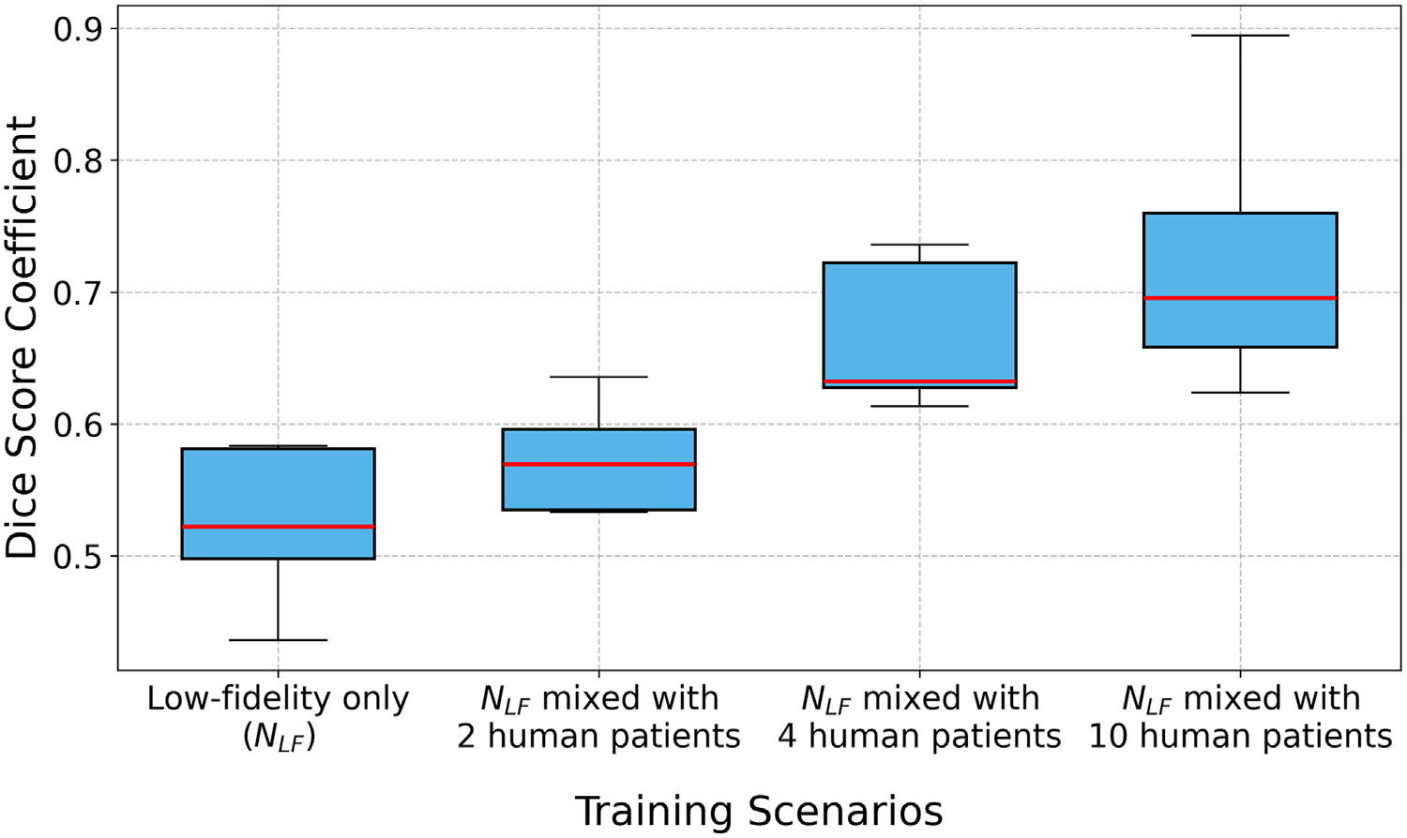
Dice similarity coefficient (DSC) scores across training scenarios: low‐fidelity data (*N*_*LF*_) only (RCCM based), *N*_*LF*_ mixed with strain data from two human patients, four human patients and finally *N*_*LF*_ mixed with strain data from nine human patients (leave‐one‐out cross‐validation approach).

We evaluated a single‐fidelity model and varying amounts of human strain data in a multi‐fidelity model, considering four different training scenarios: training with low‐fidelity data only (*N*_*LF*_), low‐fidelity data mixed with strain data from two human patients, four human patients and finally from nine human patients (**Figure** **9**). For each scenario, the DSC scores for human patients were computed and the results showed a significant increase in DSC as the number of high‐fidelity samples increased in the training data. The average DSC across five patients with single‐fidelity data alone was 0.3887, which was increased to 0.5737 and 0.6662 when two and four human patients' data were included and further improved to 0.7197 when strain data from nine human patients were used in the multi‐fidelity training as depicted in Figure 9 and Section S3 (Supporting Information). These findings highlight the importance of using simulation data and human CMR scans together to improve infarct prediction. Simulation data alone struggles to predict human infarcts accurately due to differences in data characteristics, while the scarcity of human strain data makes it difficult to rely on human data alone for accurate predictions.

## 4 Discussion

### 4.1 Non‐Invasive Identification of Infarct Regions in the LV

Our approach made use of cardiac strain data quantified directly from imaging as input features. By integrating cardiac strains with ML, we estimated infarcted tissue by extracting both local and global features. This innovative approach addressed a significant limitation of traditional methods by avoiding the need for invasive administration of GCA, reducing patient discomfort and potential risks of adverse reactions to gadolinium especially in patients with contraindications, such as renal impairment. Thus, our approach shows promise in estimating infarct regions without invasive procedures or GCA‐related concerns. Furthermore, automating the analysis eliminates inter‐observer variability due to the manual segmentation of LGE‐CMR images, which is essential for large‐scale clinical studies and reliability in patient assessments. Thus, our proposed approach holds promise to improve the speed and reliability of infarct characterization, and to make infarct estimation more accessible for routine clinical use.

From a clinical standpoint, the proposed model can be integrated into existing diagnostic workflows as a post‐processing tool applied to cine CMR‐derived cardiac strains. Since strain computation can be performed from non‐contrast images using existing registration‐based pipelines, this method does not require additional imaging or protocol changes. Once cardiac strain maps are obtained, they can be input into the ML model for automated infarct prediction. The resulting output can assist clinicians in identifying infarct regions without the need for GCAs, thereby enhancing diagnostic capabilities in standard and resource‐limited settings.

### 4.2 Rodent‐Based Simulations in Multi‐Fidelity Learning

Despite physiological differences between rodent and human hearts, rodent models remained the most widely used and well‐validated experimental systems in cardiac research because they replicated essential features of human pathology, such as myocardial stiffening, scar formation, and post‐infarct strain alterations.^[^ ^47^, ^48^ ^]^ A key advantage of rodent models was the ability to collect rich multi‐modal datasets that were not feasible in humans.^[^ ^30^ ^]^ These included invasive P‐V loop recordings, contrast‐enhanced CMR imaging, histological analysis for fiber architecture, and ex vivo biaxial tensile testing for material property estimation. This depth of data allowed for the in‐silico computational models to generate a wide range of controlled infarct scenarios in terms of size, location, and stiffness. The multi‐fidelity model then adapted this generalized knowledge using a smaller, high‐fidelity human dataset to improve prediction accuracy and clinical relevance.

### 4.3 Applicability to a Various Range of Myocardial Injuries

MI can range from mild to severe, with large variations in the extent of myocardial damage. Our multi‐fidelity model was trained using cardiac CRL strain data from a wide‐ranging dataset covering various stiffness levels representing infarct areas of different severities. By incorporating this diverse dataset of passive myocardial stiffness values into our training simulations, the model effectively learned to recognize suboptimal contraction to differentiate between healthy and infarcted regions. This capability is expected to not only perform well in severe myocardial injuries with sizable scars and substantial alterations in strain values but also be advantageous for mild MI cases, which often display abnormalities in regional strain patterns, but the injuries do not contain mature and extensive scars to be reliably detected by LGE‐CMR. Importantly, the use of rodent‐based in‐silico models enabled us to generate a wide range of infarct stiffness values that would be challenging to obtain from human data alone, due to the practical limitations of acquiring ex vivo human samples. This variability in training data enhances the model's ability to generalize across different severities of MI, ensuring reliable and efficient detection of infarct size and location in clinical settings.

### 4.4 Harnessing Image‐Based Strain Representations in Convolution‐Based Networks

A key query addressed in our research was the effective incorporation of cardiac strain data into ML models to identify infarct location. We solved this problem by converting strain data into an image‐based format rather than using numerical strain values directly. This approach was employed to utilize the capabilities of convolutional‐based networks, which are well‐known for their ability to handle pixel‐based data in tasks such as object categorization, object recognition, and scene interpretation. We harnessed the power of convolution‐based networks to examine pixel values and grasp detailed information from the complex images of strain data. Furthermore, direct utilization of numerical values of strain data was avoided in the ML model due to inconsistencies in numerical datasets from diverse CMR cases caused by the inherent variability in pixel distribution among CMR images. More specifically, we used the AHA segmentation procedure as a first step before translating the data into standardized images. This dual transformation assured uniform image sizes and spatial arrangements, which not only regularized the input features but also allowed the seamless integration of data augmentation approaches, which was a key component of model generalization. In contrast to the numerical values with tree‐based models or fully connected networks, convolution‐based networks captured relevant features directly from raw pixel values. Furthermore, this approach eliminated the need for manual feature engineering, a critical advantage that ideally aligns with the transition to image‐based strain representations.

### 4.5 Utilization of CRL Strains for Enhanced Infarct Region Identification

The trained multi‐fidelity ML model not only showed promise in the non‐invasive identification of the infarct scar sites but may also help identify injured regions of the myocardium with reduced function, such as surrounding regions closely connected with existing infarct zones. Notably, the comprehensive use of strain data enabled the potential localization of active infarct locations and the characterization of “border” zones with compromised contractility compared to the LGE‐CMR approach, which can primarily locate mature scar regions. This capability was evident in Figure 8 and Section S3 (Supporting Information), where GCA was not spread enough to locate the infarct region accurately, but our ML model still found the infarct region and assisted with estimating the extent of the border regions at risk of losing contractility, thus improving the prognosis of the transition to heart failure in MI patients. In essence, using processed cardiac strains as markers to locate infarct regions not only reduces the risk of invasive procedures by avoiding GCA injection but also promises to improve the stratification of MI at a higher risk of transitioning to systolic heart failure. Furthermore, our approach used cardiac strain data which can be obtained from more ubiquitous and affordable imaging modalities than CMR, such as echocardiography, reinforcing the possibility of implementation of such approach in clinical settings.

### 4.6 Pursuit of Best ML Model to Use Cardiac Strains

Among the single‐fidelity ML models investigated in this study, UNet exhibited comparable DSC with its variants in identifying infarct regions in human CMR strain data. However, the observed performance of UNet and its variants on human CMR is attributed to their limits in generalization across diverse datasets. All UNets demonstrated promising performance on FE simulation data (Table 4), however their challenge to effectively transition to human CMR data is noteworthy (Figure 9). The lower accuracy of these single‐fidelity frameworks on human LGE‐CMR data highlights the difficulty of transitioning trained models from synthetic data. We attempted to overcome such challenges with a multi‐fidelity ML approach. Our results indicate that for every *N*_*LF*_ value, DSC for multi‐fidelity ML approach outperformed single‐fidelity based models (Figure 9). The noted improvement after adding only a few sets of human data indicates the promise of developing accurate multi‐fidelity simulation‐based ML models with small amounts of human data, which is often difficult to obtain and process. All models, including multi‐fidelity NN, could additionally be extended to incorporate the physical laws governing the myocardial behavior,^[^ ^49^ ^]^ which could potentially augment the accuracy and also help in reducing the number of training samples required in both low‐ and high‐fidelity datasets. The more sophisticated architecture of multi‐fidelity NN could be analyzed, such as residual multi‐fidelity NN,^[^ ^50^ ^]^ with a slightly increased number of high‐fidelity realizations. This presents a potential avenue for future research, aiming to refine the performance and adaptability of these models to efficiently and reliably address the sub‐optimal strain quantification from human CMR imaging.

### 4.7 Detailed Cardiac Mechanics From 4D Cardiac Strains

Although our study focused on infarct region identification, it laid the groundwork for various possible future directions. While we exclusively provided ES strains on specific cardiac slices aligned with the AHA segmentation, it is possible to obtain complete spatiotemporal (4D) strains in the LV for training, giving our ML model richer inputs for capturing LV mechanics and locating infarct and compromised regions accurately. This expansion could constitute a significant improvement over the common utilization of cardiac strains in the form of a limited number of short‐axis slices, keeping in mind that LGE‐CMR acquisitions on short‐axis slices could, at best, locate infarct locations at those specific slices. In contrast, complete spatial strain quantification can more precisely locate infarct in the LV. Furthermore, integrating strains obtained at all time points of the cardiac cycle will open new possibilities for extracting complex insights beyond locating infarct regions. This temporal dimension allows for understanding the difference between infarct tissues not only in contractile behavior but also in relaxation and passive behaviors, assisting with determining their distinct mechanical characteristics. For instance, by analyzing the entire cycle, we may be able to distinguish between the contributions of collagen and myofibers to tissue stiffness, presenting an opportunity to improve the understanding of the passive behavior of infarct tissues.^[^ ^51^ ^]^ In essence, using 4D strains offers to improve the full characterization of myocardial scars and subsequent prognostic and therapeutic approaches in MI patients.

### 4.8 Limitations and Future Directions

Encouraged by the promise of using cardiac strains to identify infarct regions, addressing the limitations listed below can further advance the translation of our tool to the clinic:

(i) *Challenges with human LGE‐CMR validation*. Some inherent limitations of human LGE‐CMR imaging may introduce a unique set of discrepancies between the predictions by our models and those by LGE. In particular, gadolinium enhancement may not present the complete infarct location since LGE‐CMR acquisition captures infarct on selective short‐axis slices and is best at capturing mature scar tissues. In situations when the LGE distribution does not sufficiently capture the extent of infarct tissue, our model's prediction of the infarct region may differ from those by LGE‐CMR presumed to be the ground truth. Therefore, future rodent studies consisting of in vivo CMR and ex vivo infarct identification using histology can serve as a critical benchmark to evaluate and further refine our model's prediction.
(ii) *Preprocessing for strain‐based infarct identification*. Although our approach accurately estimates infarct location in both FE simulation and human datasets, it also adds a crucial intermediary step to extract strains from segmented slices, which introduces complexity before providing to the ML model. In an alternative scenario, one could envision directly utilizing the individual slices at all time points from ED to ES as inputs for our ML model. The model could potentially infer and localize infarct regions by leveraging pixel movements across time frames. While this alternative path might offer a seemingly streamlined process, it will raise a serious issue about the probable loss of subtle details necessary for precise infarct identification. In the direct utilization of imaging slices, omitting explicit strain extraction, the model may unintentionally neglect details of heart mechanics and tissue behavior that are adequately captured through strain analysis if it only considers pixel motions. As a future direction, we could further utilize strains spanning from ED to ES to capture a broader range of cardiac motion, which would better prepare the dataset for ML training, improving infarct estimation accuracy and enhancing model generalization across species.
(iii) *Modal adaptability across imaging modalities and patient variability*. We used only CMR images from a 3.0‐T clinical scanner (Siemens Verio; Siemens, Erlangen, Germany) at a single hospital. Different imaging systems and protocols may introduce subtle differences in strain measurements, which can impact the model's prediction accuracy. However, our multi‐fidelity approach is designed to incorporate cardiac strains from various imaging systems, effectively learning these differences. This adaptability has already been demonstrated in our training, as the model successfully trained and showcased its generalizability on two different datasets, including rodent and human strains. Moreover, the expanded human dataset now included both ischemic and non‐ischemic myocardial scar cases, further testing the model's generalizability across different patient pathologies (Section S3, Supporting Information). It is also important to note that our model has not yet been trained on strain data from modalities other than CMR, and future work could address this by integrating data from diverse imaging types to enhance adaptability in diverse scanner platforms and clinical environments.
(iv) *Need for age, sex, and demographically inclusive human data*. Although our model accounted for myocardial infarction across different ages and both sexes in rodents, with equal numbers of male and female subjects (n = 6 at various time points) as detailed in Section 2.2.4, it did not consider inherent differences such as age, sex, and demographic variation in the human data. These factors can significantly impact cardiac motion and may affect the model's generalizability.^[^ ^52^, ^53^ ^]^ To enhance the robustness and applicability of our method, it is crucial to incorporate human data spanning a wide range of ages and demographic groups. This would enable the model to account for the natural variability in cardiac mechanics, thereby improving its performance across diverse patient populations.
(v) *Comprehensive 3D infarct localization*. Our methodology was developed to be consistent with the cardiac imaging modalities that provide data in the form of 2D slices. Although our approach can identify infarct regions on those slices, there will be a need for an additional but straightforward extension of the method to map the full 3D extent of the infarct region, which will provide a more reliable prognostic marker than 2D representations of infarct regions. Indeed, the prediction of infarct region from cardiac strains, inherent in our method, perfectly lends itself to the possibility of 3D reconstruction of infarct regions as long as rigorous strain calculation algorithms can determine complete spatiotemporal strains through proper interpolation of cardiac motion across 2D slices.^[^ ^42^, ^43^ ^]^
(vi) *Integration of ML with multi‐scale modeling*. In our study, we successfully demonstrated the ability of ML‐based frameworks to identify the infarct region from CMR strains. However, relying solely on ML overlooks essential physical laws, leading to potentially ill‐defined problems or solutions that defy physics. Multi‐scale modeling, a well‐established approach, combines data from various scales and physical phenomena to reveal the underlying mechanisms of function. In the past two decades, this approach has proven effective in constructing detailed organ models by integrating knowledge across tissue, cellular, and molecular levels. The recent advancements in ML, with its capacity to handle diverse, multi‐fidelity data, and uncover complex correlations, offer a unique opportunity in this context. Nevertheless, multi‐scale modeling on its own often struggles to efficiently merge vast datasets from different sources and resolutions. Combining ML with multi‐scale modeling allows us to create robust predictive models that incorporate fundamental physics, address ill‐defined problems, and navigate extensive design spaces.^[^ ^54^ ^]^ Adopting a multidisciplinary approach that blends ML and multi‐scale modeling can unlock new insights into disease mechanisms, aid in identifying novel therapeutic targets and strategies, and support decision‐making processes for improving human health.

## Conflict of Interest

George Karniadakis discloses financial interests with the companies Anailytica and PredictiveIQ. Other authors declare no conflict of interest.

## Supporting information

Supporting Information

## Data Availability

The data that support the findings of this study are openly available in Zenodo at https://doi.org/10.5281/zenodo.15208063, reference number 15208063.
