# Supplementary material for: Non‐Invasive Diagnosis of Chronic Myocardial Infarction via Composite In‐Silico‐Human Data Learning
Source: Adv Sci (Weinh). 2025 Jun 19;12(30):e06933. doi: 10.1002/advs.202406933 (PMC12376577; doi:10.1002/advs.202406933)
Supplement: Supplementary file 1 — Supporting Information [file ADVS-12-e06933-s001.pdf]

ADVANCED SCIENCE
Open Access

Supporting Information

for *Adv. Sci.*, DOI 10.1002/advs.202406933

Non-Invasive Diagnosis of Chronic Myocardial Infarction via Composite In-Silico-Human Data Learning

Rana Raza Mehdi, Nikhil Kadivar, Tanmay Mukherjee, Emilio A. Mendiola, Akila Bersali, Dipan J. Shah, George Karniadakis and Reza Avazmohammadi**

Supplementary S1: Relationship between infarct size and stiffness with cardiac strains

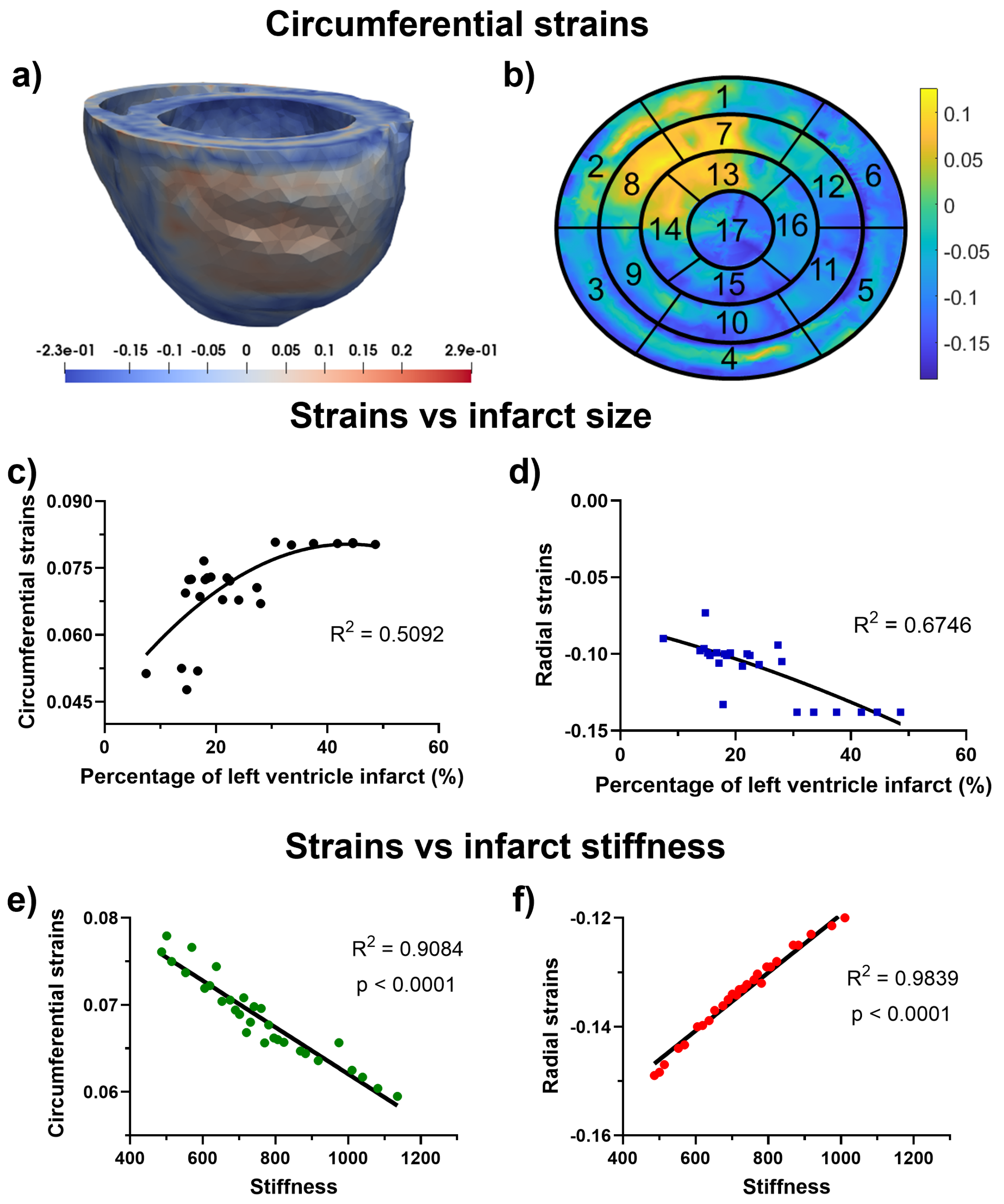

Fig. S1 a) Representative in-silico circumferential strain distribution in a biventricular finite-element heart model, b) the corresponding circumferential strains of left ventricle (LV) using the American Heart Association segmentation, c) average circumferential strain of infarcted region in the LV versus the percentage of the infarcted region of the LV, d) average radial strain of infarcted region in the LV versus the percentage of the infarcted region of the LV, e) average circumferential strain of infarcted region in the LV versus stiffness of the LV, f) average radial strain of infarcted region in the LV versus stiffness of the LV.

Supplementary S2: ML models to estimate infarct regions from low fidelity data

We applied different encoder-decoder-based designs, namely, UNet, attention UNet, dense UNet, and residual attention UNet, which are known for their precision in segmenting based on spatial variations between pixels in the image[1]. These architectures use the encoder-decoder structures to extract significant characteristics from the images, ensuring that they do not lose any important spatial information. Given this ability, applying these ML models to our low-fidelity RCCM strain data, they are expected to identify subtle changes in strain patterns that may indicate the presence of infarct, even if those changes are not readily apparent to the naked eye. Furthermore, the use of attention mechanisms in some of these architectures assists with focusing the model's attention on the most relevant parts of the image, further improving its accuracy. These encoder-decoder-based architectures are described in detail below:

UNet

UNet consists of symmetric contracting (encoder) and expansive (decoder) paths[2]. The contracting path comprises a series of convolutional layers, each followed by a rectified linear unit activation function and max-pooling layers. This arrangement progressively reduces the spatial dimensions of the feature maps while increasing the number of feature channels. This process helps the network to learn hierarchical representations of the input features, allowing it to extract relevant patterns and features. Conversely, the expansive path employs transposed convolutions to progressively upsample the feature maps, recovering spatial information and reconstructing the original input dimensions. Skip connections are employed between the encoder and decoder layers, enabling the fusion of high and low-resolution features from the encoder and decoder paths, respectively. These skip connections facilitate the precise localization of infarct regions by preserving fine-grained spatial details. The final layer of the UNet consists of a 1x1 convolutional layer followed by a sigmoid activation function. With a single-channel output, the convolutional layer condenses the multi-channel feature maps into a binary representation, where each pixel is assigned a value of either 0 or 1. The final layer predicts the probabilities of each pixel belonging to the infarct or normal region, with values closer to 1 indicating a higher likelihood of being part of the infarct and vice versa.

Attention UNet

Expanding upon the foundation of UNet, the integration of an attention mechanism with the UNet architecture[3] aims to unveil intricate, long-range dependencies crucial for enhancing infarct detection precision. While UNet effectively captures local features and spatial relationships, its reliance on convolutional operations alone may obscure subtle, interconnected patterns within the strain data. The attention mechanism serves as a valuable augmentation, introducing an additional module to scrutinize the feature map at each level. This module learns to assign importance scores to various spatial locations within the image, illuminating previously unseen regions of infarct. These importance scores guide the network to focus on the most informative strain patterns for infarct prediction. However, the advantages of attention are accompanied by computational overhead, potentially increasing model training time. Attention gates (AGs) are incorporated into the standard UNet architecture through skip connections, in which a gating vector from the decoder path is used for each pixel to determine focus regions. This gating vector includes contextual information for selectively reducing lower-level feature responses, a concept proposed in a prior study[4], which utilizes AGs for the classification of natural images. We adopted additive attention[5] to derive the gating coefficient, motivated by its higher experimental accuracy compared to multiplicative attention[6], despite its computational cost.

Dense UNet

Dense UNet incorporates dense connections, inspired by the DenseNet architecture[7], to capture a more comprehensive picture of the underlying patterns. By forming a web-like structure of layers, Dense UNet creates an interactive environment where features interact seamlessly, refining one another and extracting more meaningful information[8]. Within each dense connection layer, a 3x3 convolution is followed by batch normalization and a ReLU activation. Feature maps from preceding layers are concatenated before input into the 3x3 convolution, fostering a comprehensive information integration. Similar to the standard UNet, Dense UNet incorporates skip connections that link the encoder and decoder paths, directly copying feature maps from the contracting path to the expanding path. This inclusion of dense connections may empower the model to comprehend features effectively, facilitating the capture of even fragmented or diffuse infarcts.

Residual attention UNet

Lastly, residual attention UNet combines the strengths of two powerful techniques: residual connections and attention mechanisms for accurate infarct delineation[9]. Residual connections bypass the main data flow in the network, directly feeding the output of a previous layer to a subsequent layer. This helps to alleviate the vanishing gradient problem, leading to more stable training for deeper networks[10]. Additionally, the attention mechanism is incorporated to analyze feature maps at specific levels to guide the network's focus toward relevant information. However, utilizing the residual attention UNet carries the risk of potential overfitting, posing a challenge to the model's ability to generalize effectively to unseen data. This issue is of

utmost importance in our specific context, as the unseen data specifically involves images from human CMR. This concern is particularly crucial in our context, where the unseen data pertains to human CMR imaging.

Supplementary S3: Applicability to ischemic and non-ischemic myocardial scars

In addition to evaluating model performance across infarct severities, model's generalizability was also assessed for different scar etiologies, including both ischemic and non-ischemic myocardial injuries. While non-ischemic cases were included in the cohort, they were less represented in the training set compared to ischemic cases. Despite this, the model achieved high DSC scores across both ischemic and non-ischemic test cases during leave-one-out cross-validation. These results indicated that the model can accurately identify infarct regions across a range of underlying scar pathologies, highlighting its potential applicability in diverse clinical scenarios.

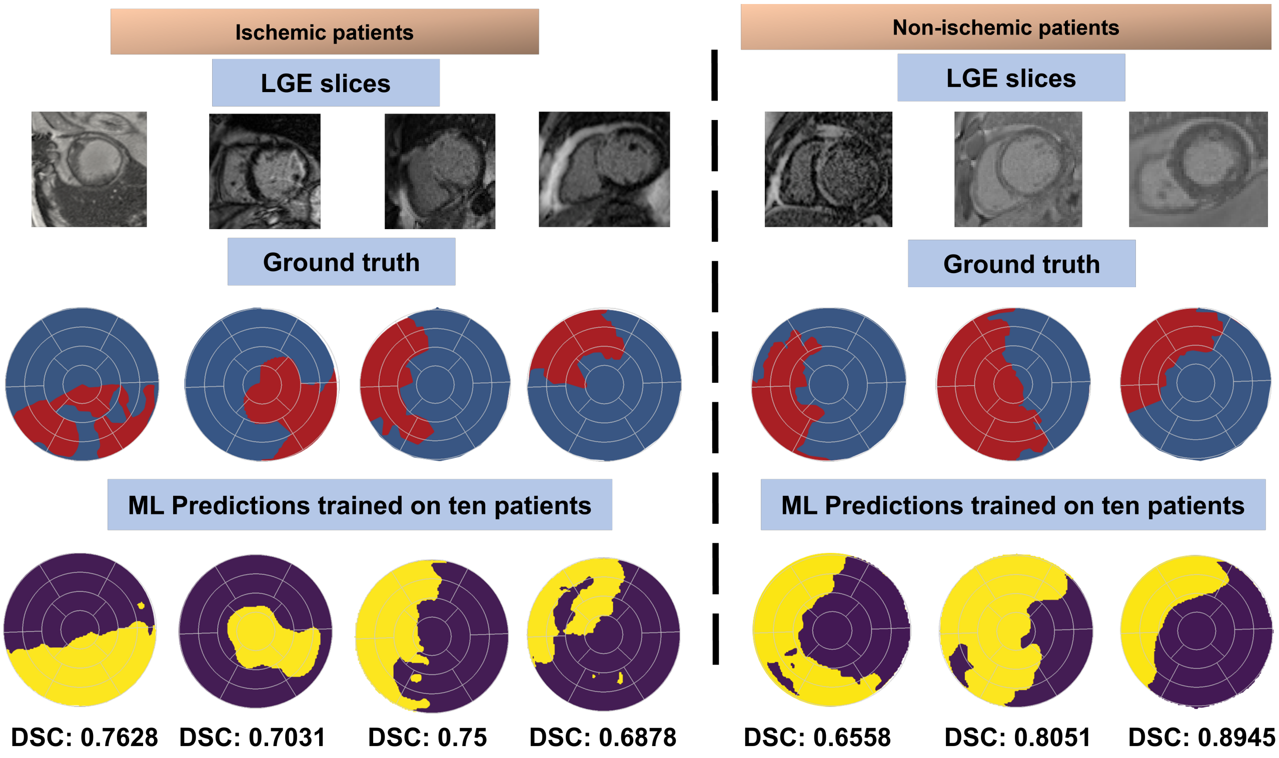

Fig. S3 Ground truth and predicted infarct regions for ischemic (left) and non-ischemic cases (right). The model was trained using leave-one-out cross-validation with nine patients in the training set and one in the test set. Ground truth infarct masks were derived from LGE-CMR (top row), and predictions (bottom row) were obtained using the multi-fidelity model trained on CRL strain data. Dice score coefficients (DSC) are shown for test cases, reflecting segmentation accuracy. Although non-ischemic cases were less represented in the training data, the model achieved consistently high DSC values across both ischemic and non-ischemic cases, demonstrating its ability to generalize across diverse myocardial scar pathologies.

Supplementary S4: Validation of Dice Score Coefficient (DSC) computation

To ensure the reproducibility of our evaluation metric, a controlled validation experiment was conducted to demonstrate how the DSC was computed in this study. Specifically, we showed that the DSC was calculated exclusively on the infarct (pathology) class within a circular mask, excluding background pixels outside the anatomical myocardium (Figure S4A). This validation was performed through two test scenarios. In the first case, the preprocessed ground truth infarct mask was compared with an identical copy of itself, yielding a perfect DSC of 1.0 (Figure S4B), confirming that the metric correctly reflects complete spatial overlap. In the second case, the same infarct mask was compared with a completely black image containing no infarct region, which produced a DSC of 0.0 (Figure S4C), verifying that background or non-pathological regions do not contribute to the DSC calculation.

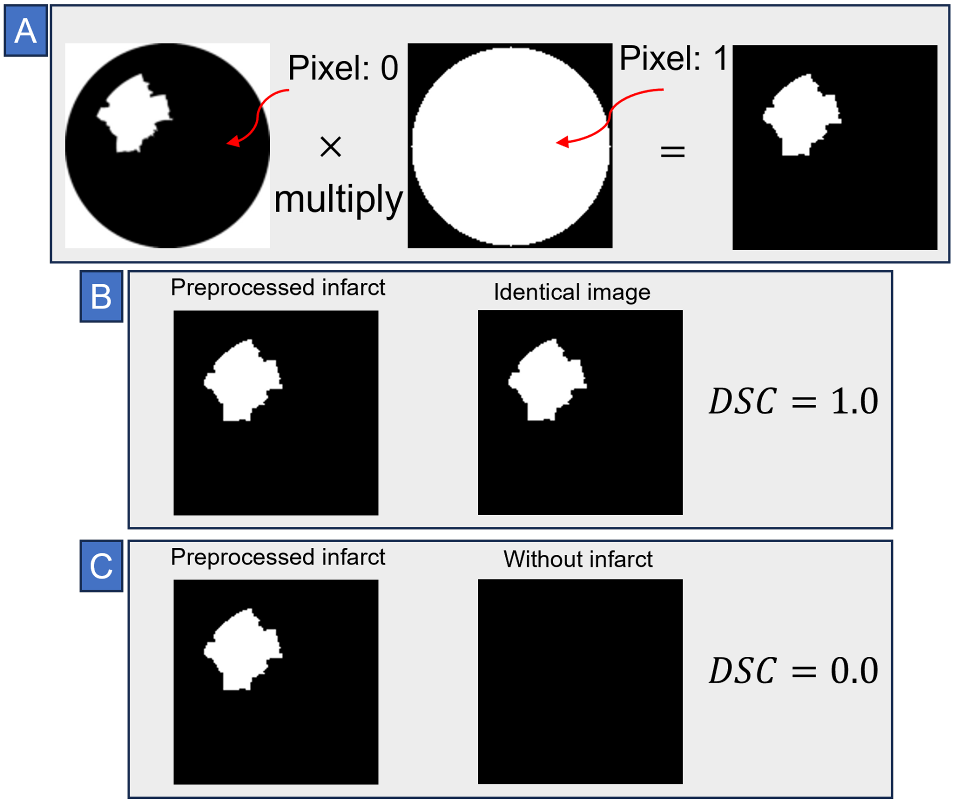

Fig. S4 Validation of Dice Score Coefficient (DSC) calculation. (A) Ground truth infarct mask multiplied by a circular mask to exclude background pixels outside the anatomical region. (B) Comparing the preprocessed infarct mask to an identical copy results in a DSC of 1.0, confirming correct overlap-based computation. (C) Comparing the infarct mask to a black (non-infarct) image results in a DSC of 0.0, confirming that background does not influence the DSC. This example is included in the Zenodo repository along with the accompanying code.

References

1. Ullah, I. *et al.* A deep learning based dual encoder–decoder framework for anatomical structure segmentation in chest x-ray images. *Sci. Reports* **13**, 791 (2023).
2. Ronneberger, O., Fischer, P. & Brox, T. U-net: Convolutional networks for biomedical image segmentation. In *Medical Image Computing and Computer-Assisted Intervention–MICCAI 2015: 18th International Conference, Munich, Germany, October 5-9, 2015, Proceedings, Part III 18*, 234–241 (Springer, 2015).
3. Oktay, O. *et al.* Attention u-net: Learning where to look for the pancreas. *arXiv preprint arXiv:1804.03999* (2018).
4. Wang, F. *et al.* Residual attention network for image classification. In *Proceedings of the IEEE conference on computer vision and pattern recognition*, 3156–3164 (2017).
5. Bahdanau, D., Cho, K. & Bengio, Y. Neural machine translation by jointly learning to align and translate. *arXiv preprint arXiv:1409.0473* (2014).
6. Luong, M.-T., Pham, H. & Manning, C. D. Effective approaches to attention-based neural machine translation. *arXiv preprint arXiv:1508.04025* (2015).
7. Zhu, Y. & Newsam, S. Densenet for dense flow. In *2017 IEEE international conference on image processing (ICIP)*, 790–794 (IEEE, 2017).
8. Chen, P., Su, X., Liu, M. & Zhu, W. Lensless computational imaging technology using deep convolutional network. *Sensors* **20**, 2661 (2020).
9. Alom, M. Z., Yakopcic, C., Hasan, M., Taha, T. M. & Asari, V. K. Recurrent residual u-net for medical image segmentation. *J. Med. Imaging* **6**, 014006–014006 (2019).

10. Milletari, F., Navab, N. & Ahmadi, S.-A. V-net: Fully convolutional neural networks for volumetric medical image segmentation. In *2016 fourth international conference on 3D vision (3DV)*, 565–571 (Ieee, 2016).
